# Enrichment and Characterisation of a Mixed-Source Ethanologenic Community Degrading the Organic Fraction of Municipal Solid Waste Under Minimal Environmental Control

**DOI:** 10.3389/fmicb.2019.00722

**Published:** 2019-04-09

**Authors:** Priscilla Carrillo-Barragan, Bernard Bowler, Jan Dolfing, Paul Sallis, Neil Duncan Gray

**Affiliations:** ^1^School of Natural and Environmental Sciences, Newcastle University, Newcastle upon Tyne, United Kingdom; ^2^School of Engineering, Newcastle University, Newcastle upon Tyne, United Kingdom

**Keywords:** bioethanol, organic municipal solid waste, mixed culture fermentation, resource recovery from waste, bacterial community composition, environmental biotechnology

## Abstract

The utilisation of the organic fraction of municipal solid waste as feedstock for bioethanol production could reduce the need for disposal of the ever-increasing amounts of municipal solid waste, especially in developing countries, and fits with the integrated goals of climate change mitigation and transport energy security. Mixed culture fermentation represents a suitable approach to handle the complexity and variability of such waste, avoiding expensive and vulnerable closed-control operational conditions. It is widely accepted that the control of pH in these systems can direct the fermentation process toward a desired fermentation product, however, little empirical evidence has been provided in respect of lignocellulosic waste substrates and different environmental inocula sources. We evaluated ethanol production from the organic fraction of municipal solid waste using five different inocula sources where lignocellulose degradation putatively occurs, namely, compost, woodland soil, rumen, cow faeces and anaerobic granular sludge, when incubated in batch microcosms at either initially neutral or acidic pH and under initially aerobic or anaerobic conditions. Although ethanol was produced by all the inocula tested, their performance was different in response to the imposed experimental conditions. Rumen and anaerobic granular sludge produced significantly the highest ethanol concentrations (∼30 mM) under initially neutral and acidic pH, respectively. A mixed-source community formed by mixing rumen and sludge (R + S) was then tested over a range of initial pH. In contrast to the differences observed for the individual inocula, the maximal ethanol production of the mixed community was not significantly different at initial pH of 5.5 and 7. Consistent with this broader functionality, the microbial community analyses confirmed the R + S community enriched comprised bacterial taxa representative of both original inocula. It was demonstrated that the interaction of initial pH and inocula source dictated ethanologenic activity from the organic fraction of municipal solid waste. Furthermore, the ethanologenic mixed-source community enriched, was comprised of taxa belonging to the two original inocula sources (rumen and sludge) and had a broader functionality. This information is relevant when diverse inocula sources are combined for mix culture fermentation studies as it experimentally demonstrates the benefits of diversity and function assembled from different inocula.

## Introduction

Research into bioethanol production from lignocellulosic biomass has focussed on agricultural (Talebnia et al., 2010; Sarkar et al., 2012; Agrawal et al., 2015) and industrial (Kádár et al., 2007; Frankó et al., 2016) products and wastes as feedstocks. Little attention has been paid to the organic fraction of municipal solid waste (OMSW). However, OMSW is an attractive substrate due to (i) its high cellulose content (Sun and Cheng, 2002; Li et al., 2012) and because (ii) it has already been discarded. The latter contrasts with feedstocks specifically produced for bioethanol production, which utilise productive agricultural land, or with feedstocks which have alternative uses such as compost (Blake et al., 2017). Furthermore, internationally there is a drive to reduce the quantities of waste going to landfill. This is of special relevance in the rapidly growing urban regions of developing countries, where overpopulation, lack of landfill space and MSW treatment/disposal associated negative costs collude (Kalogo et al., 2007; Bozorgirad et al., 2013; Leme et al., 2014).

Bioethanol production requires a complex process, capable of hydrolysing lignocellulose into cellulose and hemicellulose, and transforming the resulting mixture of sugars into ethanol (Balat, 2011). To address these challenges, numerous studies have been directed toward metabolic engineering of well-studied ethanologenic and/or industrially friendly microbial strains (e.g., *Saccharomyces cerevisiae, Zymomonas mobilis, Escherichia coli*, etc.) to expand their metabolic capabilities, aiming to improve monoculture fermentations (Hong and Nielsen, 2012; Zhou et al., 2018). Nevertheless, these processes necessarily operate under narrow closed-control conditions, are still limited and the subject of improvement (Mohd Azhar et al., 2017; Zhou et al., 2018).

An alternative approach for the sustainable production of ethanol is the application of eco-biotechnological principles (Kleerebezem and van Loosdrecht, 2007), where the production of metabolic products is achieved by employing open mixed cultures and ecological selection principles (Kleerebezem and van Loosdrecht, 2007; Temudo et al., 2008; Varrone et al., 2015). Such a mixed culture fermentation (MCF) approach is a promising alternative to enzymatic treatment and monoculture fermentation as it has shown enhancement in the conversion of cellulose to EtOH by virtue of synergistic metabolic capabilities, reducing problems such as end-product inhibition and incomplete enzyme pathways and circumvented the need to supplement high concentrations of enzymes (Kato et al., 2004; Okeke and Lu, 2011; Cheng and Zhu, 2012; Ronan et al., 2013; Oksanen et al., 2018). In addition, consortia are more resistant and resilient to perturbation, resulting in a lower requirement for process control, potentially making OMSW based mixed culture ethanol production robust and exploitable with low operational and maintenance costs (Kato et al., 2005; Okeke and Lu, 2011).

Mixed culture communities for bioethanol production should be sourced from an appropriate source environment or indeed multiple environments. This raises the question how critical the choice of a source or multiple sources might be. In addition, the comparison of the community composition arising from different inocula sources employed in MCF has been sparingly reported (Peacock et al., 2013; Jimenez et al., 2014; Simmons et al., 2014), and although some studies have mixed different inocula sources for the inoculation of MCF reactors (Temudo et al., 2008; Lin et al., 2011; Du et al., 2015), an evaluation of whether the enriched community and its function was derived from a combination of these sources has not been addressed so far. This might be due to the traditional conception that “everything is everywhere, but, the environment selects” (De Wit and Bouvier, 2006). As consequence, environmental factors, particularly pH, have been studied as major means to control the direction of fermentative metabolism (Temudo et al., 2007; Rodríguez et al., 2008; González-Cabaleiro et al., 2015). MCF studies have been performed in neutral to alkaline conditions; however, only a few have explored initially acidic media pH and showed good ethanologenic performance (Zhang et al., 2012; Ganigué et al., 2016). Fermentation at initially acidic conditions could simplify the production process, as it would facilitate the pH conditioning step considered necessary after acid-pretreatment of lignocellulosic substrates (Jönsson et al., 2013; Jönsson and Martín, 2016).

Moreover, to further simplify the production process, it has been proposed that MCF under initially aerobic conditions could not only reduce the process operational costs (Ronan et al., 2013), but also provide stability to complex microbial communities (Kato et al., 2005).

In this work, we sampled diverse environments with potential microbial lignocellulose degradation activity and incubated them with OMSW. We evaluated the resulting ethanol production and soluble fermentation profiles under either initial aerobic or anaerobic conditions and at initially neutral or acidic pH. Inocula producing the highest EtOH concentrations at the initial pH values tested were selected and combined to evaluate the ethanol production of the resulting mixed-source communities over a range of pH values. We hypothesised that a novel community enriched by mixing the previous best performing inocula could benefit from functional redundancy, allowing the mixed-source community to produce EtOH at a broader range of pH values. We also hypothesised that the enriched mixed-source community would be comprised of taxa retained from both original inocula sources and this mixed community composition would explain the putatively broader functional potential. The ultimate aim of this work was therefore to enrich and characterise a mixed-source microbial community able to produce EtOH from OMSW under minimal operational conditions, these being initially acidic pH and aerobic conditions.

## Materials and Methods

### Inocula Description and Sampling

Five different environments were sampled to enrich for bacterial communities with the diverse metabolic capabilities required to disrupt and transform lignocellulose:

(1) Compost, taken from the surface layer of a composting pile from Newcastle University’s Nafferton Farm, located 19 km west of the city of Newcastle upon Tyne on the north side of the Tyne valley (54°59′08.6″N 1°53′56.2″W).
(2) Woodland soil, taken near the river Ouseburn at Jesmond Dene from around trees where the leaf-mat was visible. Jesmond Dene is a parkland/woodland located between the areas of South Gosforth and Jesmond Vale in the city of Newcastle upon Tyne (54°59′21.6″N 1°35′26.2″W).
(3) Cow faeces, collected immediately after excretion by a cow at Newcastle University’s Nafferton Farm. The cattle at Nafferton farm are reared for milk production.
(4) Rumen from a wether sheep (Suffolk cross breed) provided by Oluwaseun Bolaji from the Department of Agriculture, Food and Rural Development at Newcastle University. Pre-slaughter, the sheep was fed a silage-based total mixed ration plus vegetables including turnips.
(5) Anaerobic digestate granular sludge from a paper industry waste water treatment facility, provided by Dr. Paul Sallis from the Environmental Engineering group at Newcastle University.

### Organic Municipal Solid Waste (OMSW) Analogue Composition, Preparation and Pre-treatment

An OMSW analogue was prepared based on the putative biodegradable categories of Mexico City’s Municipal Solid Waste (MSW), as proxy for high organics MSW of overpopulated cities in developing countries, in this case amounting to 60% of its total composition (Durán-Moreno et al., 2013). Proportions were calculated based on the organics, paper and cardboard categories as part of the OMSW. As no further information was available regarding Mexico City’s organics category composition, this component was simplified to food waste for the analogue’s preparation. Therefore, the organics category was integrated based on the proportions of food waste in United Kingdom and Wales (Quested and Johnson, 2009). Supplementary Table S1 brings the two data sources together, listing the components and proportions used in this work to prepare the OMSW analogue. All mass values in this study are reported in terms of dry weight mass.

To prepare the OMSW analogue, the spring greens, apples, bananas oranges and watermelon were purchased (Hutchinson’s Fruit and Veg Ltd., Fenham, Newcastle upon Tyne). The fruits were consumed, keeping the apple cores, banana, oranges, and watermelon peelings. The baguette, own brand egg fried rice (Tesco Stores Ltd., United Kingdom); and Butcher’s Market British meatballs pack (Iceland Foods Ltd., United Kingdom) were also purchased locally.

Using the National Renewable Energy Laboratory Analytical Procedure (Hames et al., 2008) as a guideline, each of these components was cut into small pieces and individually dried for 2 days at 55°C ± 5°C. The dried material of each food category was then individually weighed, blended into a fine powder and sieved through a 1000/μm mesh.

The paper category was produced from used office paper, sheets from magazines and newspaper, whereas the cardboard category was formed from thin, lightweight card from breakfast cereal and other packaging along with layers taken from corrugated or fluted packing board from cardboard boxes. These two fractions were also cut into small pieces and blended. Although these materials did not form a powder, their surface was disrupted and softened. The dried powdered material obtained from each food category, and the blended paper and cardboard were stored individually in polypropylene containers and kept refrigerated at 4°C until pre-treatment.

The powder material from each food component was mixed following the proportions specified in Supplementary Table S1 to form the food waste fraction of the OMSW analogue. For the diluted acid pre-treatment (Li et al., 2007), H_2_SO_4_ 1% v/v was added to the food waste mixture (3 mL acid:1 g food waste) and in separate containers, to the blended paper and cardboard fractions (4 mL acid:1 g paper/cardboard). The three individual waste categories were then incubated at 60°C overnight.

### OMSW Medium Basic Characterisation

Pre-treated OMSW analogue’s moisture (12.9 ± 0.4%), total (87.1 ± 0.4%), volatile (88.8 ± 0.5%), and fixed solids (9.8 ± 0.4%) contents were determined according to the APHA standard method 2540 (APHA, 2012) and NREL laboratory analytical procedure for moisture content (Sluiter et al., 2008).

Liquid samples taken from serum bottles containing acid and steam pre-treated mineral medium with OMSW (prepared as all microcosms in this project, see section below) were used to determine the Chemical Oxygen Demand (COD) according to the APHA method 5220 (Rice et al., 2012). The COD of the liquid fraction of the medium was 19.7 ± 0.3 g/L, equivalent to a concentration of 2,450 me^-^ eq/L. For the electron balances, COD was quantified using 8 g_COD_/e^-^ eq while mmol of end-products were converted to electron milliequivalents (e^-^ meq.) using electron equivalents per mole values (Rittmann and McCarty, 2001).

The total COD (TCOD) of the OMSW was estimated using the proportions of the general waste categories in Supplementary Table S1: 83% food waste, 10% paper and 7% cardboard, with TCOD contents (in g_COD_/g_material_) of 1.1, 0.1 and 0.1, respectively, as reported in the literature (Bayard et al., 2018) giving a TCOD content of 01.3 g_COD_/g_OMSW_. As the input per bottle was 2.5 g_OMSW_/50 mL_medium_ (see microcosms preparation), the estimated TCOD content of the medium if all the OMSW dissolved would be 65 g_COD_/L equivalent to 8125 me^-^ eq/L. Electron balances were calculated as described for the COD of the liquid fraction.

### Experimental Design

The initial experimental design to test the effects of inocula source, initial oxygen presence and initial pH on EtOH production, was divided into two independent set ups, each one corresponding to incubations under initially neutral or acidic pH (see Supplementary Figure S1). Both set ups considered two factors: (A) Inocula source and (B) Initial oxygen conditions.

At initially neutral pH, factor (A) had 8 levels, the 5 inocula sources (compost, woodland soil, cow faeces, rumen, and sludge), plus microcosms inoculated with a mixture of all original sources (henceforth named “All”), inocula only control (“IOC”, microcosms with the mixture of all inocula without carbon source) and substrate only control (“SOC,” microcosms with media and OMSW analogue, without inocula added). Factor (B) had 2 levels, namely, initial anaerobic or aerobic conditions. The 16 possible combinations (A^∗^B) were each tested in triplicate with exception of the substrate only control which was run in duplicate. A total of 46 microcosms were thus prepared. To narrow down the inoculum selection for subsequent analysis, at initially acidic pH, the set up described above was repeated excluding the two inocula with the lowest EtOH production observed. The 12 possible combinations were tested in triplicate. These microcosms were incubated at an initial pH of 5.5, the approximate pH of the substrate after acid pre-treatment. To evaluate the effect of initial pH on EtOH production, the results of maximal EtOH production by the inocula tested in both experiments were statistically compared as described below.

The previously described experiments results informed the experimental design for the subsequent independent test (see Supplementary Figure S2), in which two factors were considered: (A) Inocula source and (B) Initial pH. Factor (A) had 4 levels, rumen, sludge and the mixture of both (R + S), plus substrate only control (SOC). Factor (B) had 3 levels, initial pH 4.5, 5.5 and 7.0. Substrate only controls (SOC) were only prepared for pH 4.5 and 7. The 11 possible combinations were tested in triplicate.

### Microcosms Preparation and Set Up

In line with the objective of this study of achieving low-control EtOH production, RM medium (Ronan et al., 2013) containing urea (2 g/L), KH_2_PO_4_ (2 g/L), K_2_HPO_4_ (3 g/L), yeast extract (2 g/L), trace mineral solution 1:100 (v/v), and resazurin (0.002 g/L), was modified, replacing urea and yeast extract with the acid pre-treated OMSW analogue fractions (83% food waste, 10% paper, and 7% cardboard). 2.5 g of the acid pre-treated OMSW analogue (2 g of food waste, 0.3 g paper, and 0.2 g cardboard) were placed into 120 mL serum bottles, after which 50 mL of modified RM media were added to each of these (5% organics medium).

The bottles were autoclaved at 121°C for 15 min for the double purpose of sterilising the culture media, and completing the OMSW analogue pre-treatment, as it has been shown that the combination of diluted acid and steam pre-treatments is more effective for the disruption of lignocellulosic materials (Li et al., 2007).

After autoclave sterilisation and before inoculation, the pH of the medium was measured using a Mettler Toledo^TM^ FiveEasy^TM^ Plus FP20 pH metre, fitted with an InLab pH electrode, and adjusted, if necessary, according to the experimental design with NaOH 1 M or H_2_SO_4_ 1 M. No further pH adjustment was made after inoculation. It must be noted that the addition of some of the inocula had an effect on the initial pH of the medium, and the pH values reported in this study for day 0 are those measured after inoculation.

Each bottle was then inoculated with 2.5 g of the corresponding inoculum, manually agitated and initial samples were taken. Microcosms prepared to test possible mixed-source ethanologenic enrichments (“all” and R + S) in the different experiments were inoculated with equivalent mass proportions of each of the corresponding inocula adding up to 2.5 g (e.g., 1.25 g of each, rumen and sludge were used as inoculum for the R + S replicates). The bottles were then sealed with butyl rubber stoppers and aluminium seal crimp caps. All experimental set ups were incubated in the dark, at room temperature (approximately 20°C) under static conditions.

### GC-FID Quantification of Soluble Fermentation Products

Soluble fermentation products (EtOH, butanol and acetic, propionic and butyric acids) were quantified using a gas chromatography with flame ionisation detection method (GC-FID). A Trace GC Ultra from Thermo Scientific coupled with an automatic injector was used. The injection volume was 1 μL, and to prevent contamination of the capillary column the injection port was fitted with a glass liner (5-mm i.d.), appropriate for split analysis (split ratio 30:1). An Agilent J&W HP-INNOWAX column coated with polyethylene glycol (30 m × 0.25 mm × 0.25 mm) was used. The temperature of the FID was 220°C, and the injector temperature was 220°C. Hydrogen was the carrier gas at a flow rate of 1.5 ml/min at 55 kPa, running at 35°C (5 min)-150°C/5(0)-250/20(2), for 38 min per sample. Solutions of ASC reagent grade ethanol, butanol, acetic, propionic and butyric acids were prepared in distilled water at concentrations of 0, 2, 4, 8, 15, 30, and 60 mM. These solutions were then analysed in triplicate for the construction of calibration curves at the beginning of each experiment. For quality control, freshly prepared standard solutions of EtOH and a mixture of volatile fatty acids (VFAs) were prepared and quantified at the start and end of each run of GC-FID analysis. Liquid samples from each experiment were centrifuged at 13,300 rpm for 3 min. 1 mL from these was then filtered using a hydrophilic 0.2 μm pore sterile Millipore philtre. Filtered samples were stored in a GC glass vial and kept at 4°C until analysis on the same day of sampling.

### Statistical Analysis of Physicochemical Data

The R programming language and free software R version 3.4.3 (2017-11-30) – “Kite-Eating Tree” was used to conduct statistical analyses of physicochemical data. The quantitative comparison of the effects of the different variables tested and their possible interactions on EtOH production was done through 2-way ANOVA analyses. To assess if the data met the 2-way ANOVA test assumptions, the homogeneity of variances and normality of the residuals were examined through the Levene’s test for homogeneity of variance and the Shapiro–Wilk normality test. When the 2-way ANOVA analysis was valid and significant (*p*-value < 0.05), a Tukey HDS test was conducted to do multiple pairwise-comparisons between the means of groups and determine which of them were significantly different.

Correlation analyses were also computed to evaluate the relationship between pH and inocula source in EtOH production. To assess the validity of the correlation analyses, the linear relationship of the continuous variables and normal distribution of the data sets were examined through linear regression and the Shapiro–Wilk normality test. As the data of one or more variables in each data set was not normally distributed, the non-parametric Kendalls’s rank correlation tau was done for consistency in the analyses. The alternative to the null hypothesis was that true correlation coefficient is not equal to 0 at the 95% confidence level.

### Molecular Biology Analyses

#### DNA Extraction

Due to the heterogeneity of the samples containing a high number of solid particles, the PowerSoil^®^ DNA Isolation Kit (Mo Bio Laboratories Inc., United Kingdom) was used to extract genomic DNA from 0.5 mg of samples taken from microcosm listed in the Supplementary Table S2 according to the manufacturer’s protocol. A procedural blank was included.

#### Ion Torrent PGM Sequencing Pooled Library Preparation

For bacterial community analyses, DNA extracts were subjected to PCR amplification using the universal primers F515 and R926 encompassing the V4–V5 region of the 16S rRNA gene (Quince et al., 2011). Following the manufacturer’s guidelines (Life Technologies, 2012), fusion primers were used. Hence, the F515 primer included an ‘A’ adaptor (25 bp,5′-CCATCTCATCCCTGCGTGTCTCCGACTCAG-3′) followed by a unique Golay barcode per sample (12 bp) and a key spacer (3 bp, 5′-GAT-3′) at the 5′ end for error correcting (D’Amore et al., 2016). Primer R926 was ligated to a ‘B’ truncated P1 adaptor (25 bp, 5′-ATCACCGACTGCCCATAGAGAGG-3′) attached to the 5′ end. Adding up to a 481 bp amplicon size. Details of the Golay barcodes used per sample can be found in the Supplementary Table S2.

A Bibby Scientific Techne TC-512 gradient thermal cycler was used to conduct the amplification. The PCR programme consisted of 4 min of initial denaturation at 95°C followed by 30 cycles of 1min denaturation at 95°C, 45 s for annealing at 55°C and 1 min of extension at 72°C. The final extension was done for 10 min at 72°C. The amplified libraries were purified using an Agencourt Ampure XP purification Kit (Beckman Coulter Inc., United Kingdom). The manufacturer’s protocol was followed. After which an equimolar pool of the amplicon libraries was prepared. The pooled library was submitted to the Environmental Engineering research laboratory, Newcastle University for sequencing with the Ion Personal Genome Machine^®^ (PGM^TM^) System using an Ion 316^TM^ Chip v2 (Life Technologies, United Kingdom). This Targeted Locus Study project has been deposited at DDBJ/EMBL/GenBank under the accession KCKJ00000000. The version described in this paper is the first version, KCKJ01000000.

#### 16S rRNA Sequencing Processing and Community Composition Analyses

The Mothur v.1.39.5 software (Schloss et al., 2009) was used to quality process and cluster sequences with a minimum length of 275 nt (from 6,96,397 high quality reads) into OTUs (97% similarity) as described by Schloss et al. (2011), in the 454-SOP. All libraries were rarefied to the smallest library count (8,987 reads). The OTU table and associated files generated were then used to conduct ecological and ordination analyses.

The OTUs/abundance rarefied data was imported into PRIMER 7 software (Primer-E Ltd., Plymouth, United Kingdom) to compute the Bray Curtis similarity matrix between each rarefied sample. The Bray–Curtis similarities were then used within PRIMER 7 to produce an nMDs ordination plot. A non-parametric ANOSIM test was used to evaluate the statistical significance (0.1% significance level of sample statistic) of community composition differences between groups of samples. The most frequent and abundant OTUs for each group of samples defined by the ANOSIM test, were determined using the similarity percentages (SIMPER) test on the Bray–Curtis similarity matrix indices. Before the resemblance matrix computation, the data set was standardised, and square root transformed. Additionally, under the assumption that the most abundant OTUs are the most active within a community, the transformed data set was filtered (Clarke and Gorley, 2015) to only consider OTUs accounting for >1% percent of the total number of individuals in each sample.

Additional to the taxonomy assignments (up to genus level) for the OTUs made by Mothur [using the Silva database release 128 (Quast et al., 2013)], the most abundant representative sequences within each OTU were aligned against the 16S RNA sequences (Bacteria and Archaea) GenBank database using the megablast algorithm (Boratyn et al., 2012).

## Results

### Effect of Initial pH and Inocula Source on EtOH Production From OMSW

**FIGURE 1:**
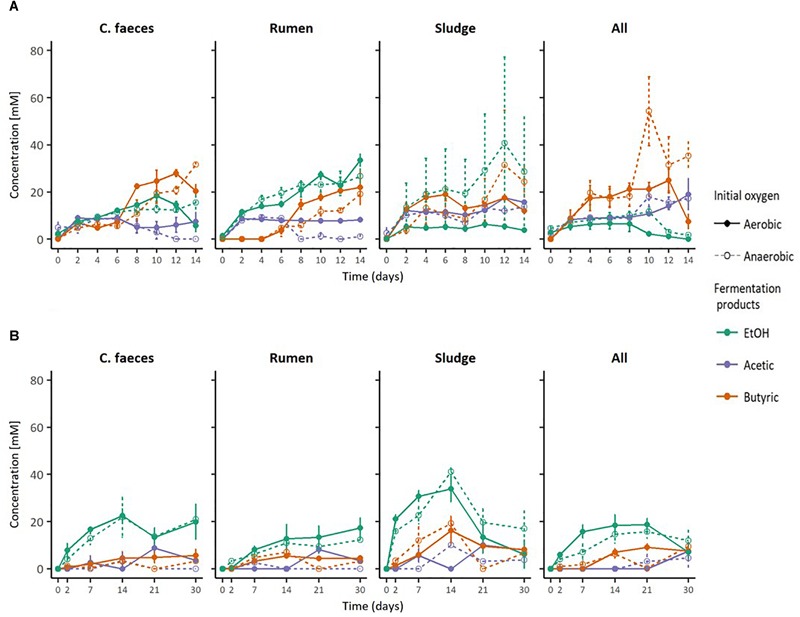
Fermentation products by the different inocula tested under initial **(A)** neutral and **(B)** acidic pH. The error bars represent the standard error of the mean (SE), *n* = 3.

The conversion of an acid pre-treated OMWS analogue by different inocula to soluble fermentation products when incubated under initially anaerobic or aerobic conditions at initially neutral or acidic pH was studied and compared (Figure 1). After 2 days of incubation, EtOH production was observed in all microcosms, however, its dominance within the fermentation products quantitatively varied in relation to the initial pH and inocula source.

At an initially neutral pH (6.70 ± 0.04), the rumen inoculum significantly produced the highest EtOH concentrations (2-way ANOVA with Tukey HDS *post hoc* analysis, *p*-values < 0.05. See Supplementary Table S3 for details), with EtOH being the dominant end-product. In contrast, the main soluble fermentation product of the rest of the inocula was butyric acid (see Table 1). When comparing incubations under initially aerobic or anaerobic conditions for any of the inocula there was no significant difference in their maximal EtOH production (i.e., rumen-aerobic vs. rumen-anaerobic *p*-value 0.927). See Supplementary Table S3 for details on the relevant results of Tukey HDS analysis. No fermentation activity was observed either in the inocula only or substrate only controls.

**Table 1:** Maximal concentrations of measured fermentation products by each inocula tested.

Inoculum	In. pH	In. O_2_	Fermentation products [mM]_max_				
			EtOH	BuOH	Acetic	Butyric	Propionic
C. faeces	Neutral	-	15.6 ± 3.2	2.28 ± 0.6	0.5 ± 0.5	31.7 ± 1.2	1.3 ± 0.4
		+	18.4 ± 2.3	3.8 ± 0.8	9.0 ± 0.3	28.0 ± 1.4	1.2 ± 0.0
	Acid	-	21.0 ± 6.5	ND	3.4 ± 3.3	3.4 ± 0.3	1.3 ± 0.7
		+	22.6 ± 2.8	2.8 ± 1.6	8.8 ± 1.7	5.7 ± 2.8	ND
Rumen	Neutral	-	26.8 ± 3.2	1.3 ± 0.7	8.9 ± 0.2	19.2 ± 1.9	ND
		+	33.6 ± 2.8	2.2 ± 0.2	8.7 ± 0.3	21.9 ± 7.5	1.1 ± 0.6
	Acid	-	12.3 ± 1.2	10.9 ± 6.7	2.9 ± 2.7	7.1 ± 1.2	4.6 ± 0.4
		+	17.3 ± 4.4	35.5 ± 20.1	8.2 ± 1.1	5.4 ± 0.9	ND
Sludge	Neutral	-	28.6 ± 23.6^∗^	2.1 ± 0.2	13.5 ± 0.9	31.5 ± 23.3	8.5 ± 2.2
		+	6.3 ± 1.2	2.4 ± 0.1	17.6 ± 0.2	18.9 ± 2.6	7.1 ± 0.5
	Acid	-	41.2 ± 1.6	8.3 ± 1.3	10.0 ± 0.2	19.2 ± 0.9	6.8 ± 0.6
		+	33.3 ± 5.6	4.8 ± 0.7	10.0 ± 1.6	16.3 ± 5.9	0.4 ± 0.3
All	Neutral	-	11.7 ± 1.2	2.4 ± 0.5	10.9 ± 0.3	21.2 ± 2.9	2.2 ± 0.2
		+	2.2 ± 0.6	2.4 ± 0.4	19.1 ± 6.9	25.1 ± 4.7	10.5 ± 0.6
	Acid	-	15.7 ± 2.8	2.6 ± 1.3	4.6 ± 4.6	9.5 ± 0.2	12.8 ± 1.4
		+	18.8 ± 2.5	3.2 ± 0.5	7.6 ± 3.8	7.6 ± 0.4	0.7 ± 0.4
Compost	Neutral	-	11.6 ± 1.6	3.0 ± 0.2	24.2 ± 2.5	31.4 ± 4.2	1.9 ± 0.3
		+	9.6 ± 1.7	2.7 ± 0.2	24.5 ± 0.5	22.5 ± 6.1	1.2 ± 0.0
W. soil	Neutral	-	10.0 ± 1.3	6.0 ± 0.4	23.8 ± 1.3	52.7 ± 11.1	3.1 ± 0.6
		+	11.0 ± 0.9	5.0 ± 0.8	17.5 ± 0.4	28.6 ± 7.7	1.5 ± 0.2
Substrate only control	Acid	-	8.7 ± 3.8	ND	ND	ND	ND
		+	4.5 ± 4.0	ND	ND	ND	ND

*In., Initial; [mM]_*max*_, fermentation product maximal concentration produced by the individual inoculum; ND, Not detected. ^∗^High [EtOH]_*max*_ is due only to one replicate (n = 3), if n = 2, [EtOH]_*max*_ = 4.40 mM ± 1.62 mM*.

At an initially acidic pH (5.33 ± 0.02), EtOH was the major soluble fermentation product of all the inocula tested (Figure 1B). However, at this initial pH sludge produced significantly the highest EtOH concentration (2-way ANOVA with Tukey HDS *post hoc* analysis, *p*-values < 0.05, Supplementary Table S3) after 14 days. In these experiments the woodland soil and compost inocula were omitted but incubation times were increased to 30 days (see experimental design for justification). As for the neutral pH experiment, no fermentation activity was observed in the inocula only controls. However, EtOH was produced by the substrate only controls, under both, anaerobic and aerobic conditions (Table 1), after 14 and 21 days of incubation, respectively, suggesting potential contamination after autoclaving or the possible enrichment of fermentative microorganisms from the acid pre-treated OMSW analogue. No other fermentation products were detected in the controls. As for the neutral pH incubations, the initial oxygen conditions did not have a significant effect in the maximal EtOH production reached by the different inocula (Supplementary Table S3).

In a statistical comparison between the initially neutral and acidic experiments (2-way ANOVA and Tukey HDS *post hoc* analysis) maximal EtOH concentrations were overall significantly different (*p*-value 0.044). However, the maximal EtOH production by the rumen at an initial neutral pH was not significantly different to that of sludge under initially acidic conditions (*p*-value 0.159).

Likewise, general comparisons of the maximal total productivity (summative soluble end-products) by rumen and sludge were done (2-way ANOVA, 95% confidence level). The total productivity of rumen at neutral pH (185.6 ± 38.9 solubleC meq./L, 976.2 ± 197.9 e^-^ meq./L) and sludge at acidic pH (202.3 ± 6.8 solubleC meq./L, 711.2 ± 58.79 e^-^ meq./L) in terms of carbon and electron equivalents were not significantly different (*p*-value 0.060). However, the proportion of EtOH from these total maximal production values by rumen (38.5 ± 4.8% solubleC meq./L, 43.2 ± 5.0% e^-^ meq./L) were about 10% lower than those of sludge (48.5 ± 3.9% solubleC, 58.7 ± 3.4% e^-^ meq/L), suggesting initial acidic pH moderately drives mass and electron balances toward EtOH production.

In average, about 10.4% of electrons estimated to be initially fed as OMSW (TCOD) were recovered in both systems (rumen and sludge). While 34.4% of electrons in the liquid fraction of the medium (COD) ended up as soluble fermentation products, from which EtOH represented more than 50%. These results suggest that hydrolysis of the solid material seems to be the bottle-neck of the process and future work should look at the biodegradable methane potential of the OMSW before and after pre-treatment.

### Analysis of pH and Maximal EtOH Production in Ethanologenic Incubations

**FIGURE 2:**
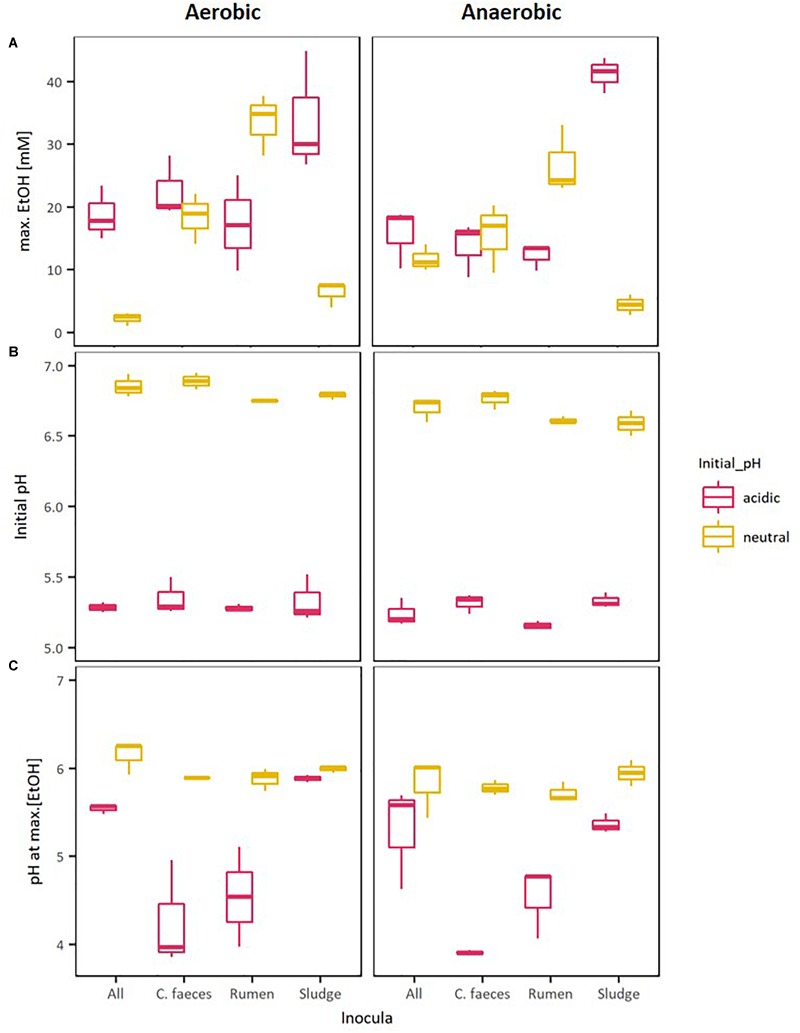
Boxplots of: **(A)** maximal EtOH production by the different inocula tested in initially acidic and neutral pH, **(B)** their initial pH, and **(C)** their pH at max. EtOH concentrations.

Figure 2 provides a direct comparison of the maximal EtOH concentrations reached in the previous experiments together with the corresponding initial pH and pH of the medium when maximal EtOH concentration was observed. The data shows a clear separation between the initial pH of the neutral (pH∼6.8) and acidic (pH∼5.3) treatments. However, disregarding their apparent optimal initial pH for ethanologenesis, pH values of rumen and sludge inoculated microcosms measured on the day of their corresponding maximal EtOH production were not significantly different (pH∼6. 2-way ANOVA with Tuckey HDS *post hoc* analysis, *p*-value 0.722).

To further evaluate whether differences observed in the performance of the inocula tested in these experiments were related to the pH of the medium, Kendalls’s tau non-parametric correlation rank (maximal EtOH concentrations, initial pHs and pHs at maximal EtOH) of combined replicate data from both aerobic and anaerobic data sets were computed (Table 2).

**Table 2:** Correlation analysis results of maximal EtOH production, initial pH, and pH at maximal EtOH concentration.

	Initial pH-max		pH at max		Initial pH-pH at	
Inocula	[EtOH]		[EtOH]-max [EtOH]		[EtOH]_max_	
	τ	*p*-value	τ	*p*-value	τ	*p*-value
All	-0.455	0.045	-0.412	0.063*	0.565	0.011
C. faeces^∗∗^	0.061	0.840*	0.078	0.730*	0.791	0.000
Rumen	0.708	0.002	0.626	0.005	0.657	0.003
Sludge	-0.600	0.01	-0.564	0.017	0.382	0.121*

*τ, Kendalls’s τ. *^∗^Not significant at 95% confidence level. ^∗∗^Data did not meet the linear covariation assumption for correlation test validity*.*

Significant correlations between maximal EtOH concentrations and initial pH were found, but also with the pH of the medium at the day of maximal EtOH production for the rumen and sludge inocula. Remarkably, rumen had a positive correlation with both initial and pH at [EtOH]_max_, whereas sludge had a negative correlation with initial pH and pH at max [EtOH].

These results revealed that similar ethanologenic activity could be achieved at either initial acidic and neutral pH values when using different inocula sources, namely rumen and sludge, leading to the hypothesis that a novel community enriched by mixing these inocula sources could benefit from specialised functional redundancy, allowing EtOH production at a range of pH.

### Ethanologenic Activities of Single and Mixed-Source Communities Over a Range of Initial pH

**FIGURE 3:**
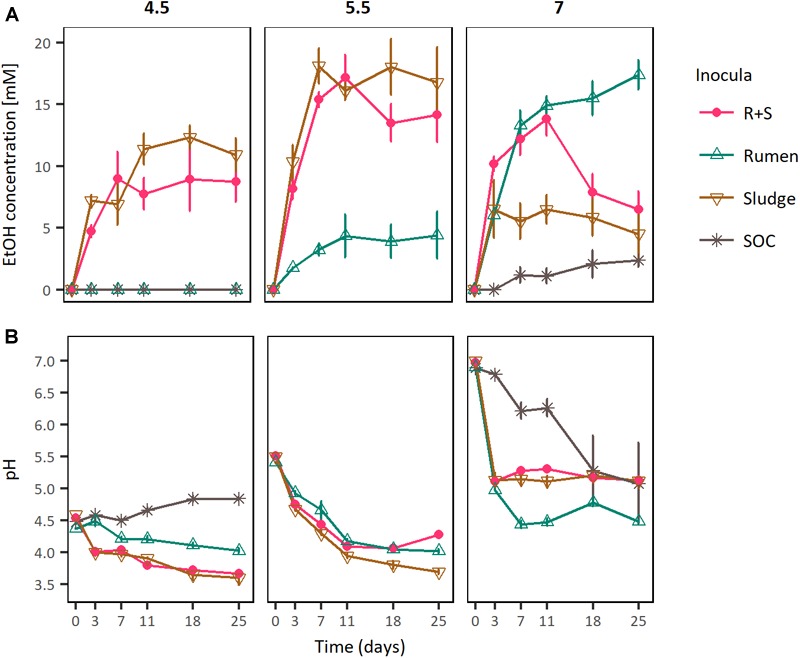
**(A)** EtOH production profiles and **(B)** pH curves of rumen, sludge, and R + S incubated at a range of initial pH. SOC, substrate only control at initial neutral pH. The error bars represent the standard error of the mean (SE), *n* = 3.

To test the specialised functional redundancy hypothesis and more generally to evaluate the reproducibility of the different inocula responses observed in the previous experiment, microcosms amended with OMSW were inoculated with rumen or sludge or a mixture of both sources (R + S) and incubated at initial pH values of 4.5, 5.5, and 7.0 (Figure 3).

In these experiments the maximal ethanologenic activities of the sludge and R + S incubations were not significantly different to each other when incubated at initial pH values of both 4.5 and 5.5. However, they were significantly different at an initial pH of 7, where notably, the R + S maximal EtOH production was not significantly different to that of the rumen incubations. Whereas, the sludge inoculated microcosms EtOH productivity was significantly lower and similar to that of the substrate only control (Supplementary Table S4).

Comparing ethanologenesis across these experiments rumen maximal ethanologenic activity at pH 7 (17.39 mM ± 1.18 mM) was not significantly different to that of sludge (18.03 mM ± 2.24 mM) or R + S (17.19 mM ± 1.82 mM) at pH 5.5. Additionally, EtOH producion by R + S under initial pH 5.5 and 7 was also not significantly different (*p*-value 0.902), supporting the hypothesis that a combination of rumen and sludge inocula leads to a robust community able to produce EtOH at a broad range of pH values.

### Rumen, Sludge and R + SBacterial Communities’ Compositionand Ordination

After quality processing 31,356 OTUs were identified among all samples and inocula only controls. The t_0_ rumen library had the richest (3,190 ± 37 OTUs), most diverse (159 ± 10) and most even (0.1 ± 2.6e-3) bacterial community among the inocula, while the t_0_ sludge richness (2,022 ± 58 OTUs), diversity (12 ± 1.4) and evenness (5.7e-3 ± 5.4e-4) were the lowest. Initially, R + S showed high richness (2,673 ± 146 OTUs), but low diversity (34.2 ± 12) and evenness (1.2e-2 ± 3.6e-3). The inocula controls were comparatively less rich (1,810 ± 15 OTUs), but were highly diverse (207 ± 13) with even bacterial composition (1.1 ± 7.9e-3).

After 11 days, the richness of all the communities had dramatically drecreased (53% for rumen, 40% for sludge and 34% for R + S) as well as their respective diversity and evenness indices. Rumen had the least diverse (6.6 ± 0.5) and least even (4.4e-3 ± 2.1e-4) bacterial composition, while R + S richness (1775 ± 138 OTUs), diversity (14.4 ± 2.6), and evenness (8.0e-3 ± 8.9e-4) although variable, were the highest among the inocula. Sludge remained the least rich (1207 ± 58 OTUs) community, but showed higher diversity (7.1 ± 1.1) and evenness (5.8e-3 ± 6.4e-4) than rumen, with values that remained relatively constant with those obtained for *t*_0_. The substrate only blank bacterial composition indices also decreased notably (48%). The decrease in richness and diversity in all inocula suggests the enrichment of OTUs fitted to grow under the specific initial pH conditions and utilise OMSW as substrate.

**FIGURE 4:**
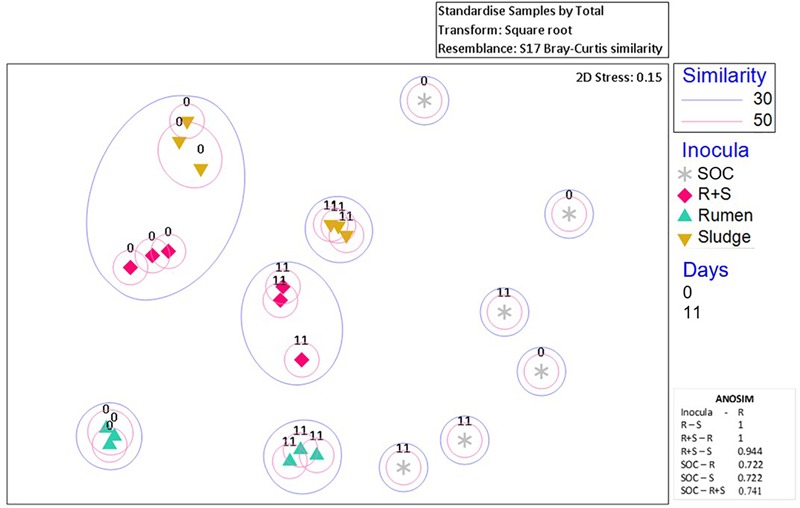
nMDS plot of incubation communities at the time zero (denoted by “0”) and the time of maximal EtOH production (denoted by “11”). Blue or pink circles encompass communities which share 30 and 50% similarities, respectively. The 2D stress value indicates good representation of the data on these 2D nMDS plots. ANOSIM R values (0.1% significance level) range from 0 to 1 where 1 = high dissimilarity between clusters. R, rumen pH 7; S, sludge pH 5.5; R + S, mixed-source community pH 5.5; SOC, substrate only control.

Additionally, nMDS ordination plots of the day 0 microcosms and after 11 days enrichment revealed distinct community composition differences between the inocula inocula (Figure 4). Although the sludge and R + S *t*_0_ samples shared at least 30% similarity, after 11 days of enrichment, each set of replicates for the individual inocula ordinated close together.

The SOC bacterial enrichment indicates that after autoclaving there was potential for contamination. However, the community compositions in the substrate controls were highly variable across sampling times and replicates, appearing to be completely independent of each other, which could also suggest the autoclave and pre-treatment survival and subsequent enrichment of bacterial communities randomly derived from the OMSW waste. Regardless of this contamination or survival of indigenous organisms the authors do not consider this to have had a major impact on the community composition and function of the environmental inocula as the lag phase for this activity was so much longer than observed in the inoculated microcosms.

SIMPER analysis (see section “Materials and Methods”) was conducted to identify the most abundant OTUs which most characterised each group of treatment replicates (Table 3) i.e., those that cumulatively contributed to at least 70% of the total similarity between groups.

**Table 3:** Most frequent and abundant OTUs (genus level) found in each inocula group at the beginning of the experiment and after 11 days of enrichment as determined by a SIMPER test.

OTU	Identity	Sim/*SD*	Contr%	OTU	Identity	Sim/*SD*	Contr%
**Rumen day 0**				**Rumen 11 days**			
Average similarity: 83.62				Average similarity: 84.48			
Cumulative similarity: 74.75				Cumulative similarity: 71.35			
13	*Ruminococcus*	17.95	11.78	2	*Pseudomonas*	21.74	31.92
14	*Millionella*	8.08	10.37	4	*Aeromonas*	11.06	20.66
19	*Lutispora*	20	9.63	12	*Carnobacterium*	44.07	6.9
23	*Prevotella*	17.35	8.61	10	*Enterococcus*	8.7	6.37
20	*Succiniclasticum*	21.75	8.61	5	*Leuconostoc*	7.05	5.5
34	*Holdemania*	17.7	7.14	–	–	–	–
41	*Victivallis*	8.73	6.7	–	–	–	–
37	*Spiroplasma*	7.95	6.36	–	–	–	–
65	*Paraprevotella*	6.58	5.57	–	–	–	–

**Sludge day 0**				**Sludge 11 days**			
Average similarity: 86.29				Average similarity: 88.04			
Cumulative similarity: 70.65				Cumulative similarity: 72.44			
1	*Pseudomonas*	23.69	31.02	3	*Clostridium*	38.07	21.85
7	*Lentimicrobium*	12.37	17.7	9	*Pseudomonas*	35.25	15.7
6	*Proteiniphilum*	21.67	11.11	5	*Leuconostoc*	41.32	14.01
16	*Clostridium*	19.89	5.61	1	*Pseudomonas*	7.5	10.81
8	*Acinetobacter*	3.53	5.21	11	*Macellibacteroides*	10.62	10.06

**R + S day 0**				**R + S 11 days**			
Average similarity: 77.03				Average similarity: 81.93			
Cumulative similarity: 71.67				Cumulative similarity: 71.89			
1	*Pseudomonas*	3.53	17.82	3	*Clostridium*	57.48	14.66
6	*Proteiniphilum*	4.22	12.51	10	*Enterococcus*	146.71	11.62
13	*Ruminococcus*	34.51	7.68	8	*Acinetobacter*	52.91	9.55
7	*Lentimicrobium*	5.91	7.58	12	*Carnobacterium*	12.88	8.91
21	*Leptolinea*	9.82	4.93	1	*Pseudomonas*	14.91	7.1
15	*Paludibacter*	50.93	4.84	2	*Pseudomonas*	3.48	6.85
14	*Millionella*	10.88	4.68	5	*Leuconostoc*	2.37	6.67
24	*Mucinivorans*	65.25	4.68	4	*Aeromonas*	18.41	6.53
16	*Clostridium*	6.36	3.84	–	–	–	–
11	*Macellibacteroides*	2.35	3.12	–	–	–	–

*Contr%, an OTU’s individual contribution to the average composition of each inoculum/sampling day group in descending order. Sim/SD, the ratio of the average contribution divided by the standard deviation of those contributions across all sample pairs in each group (Sim/SD). This metric allows the identification of OTUs that contributed consistently to the average bacterial composition of each group of replicates*.

The SOC initial average similarity (43.97%) was mostly due to five OTUs, three of which were not taxonomically assignable below the bacterial domain level, This diversity decreased (32.29%) by 11 days and was characterised by three sequence types all closely related to *Leuconostoc mesenteroides* (>97% similarity), a facultative anaerobe typically found on the skin of fruits and vegetables (Lampert et al., 2017) and two *Pseudomonas* sequence types related (99% similarity) to *Pseudomonas proteolytica* and *Pseudomonas weihenstephanensis*, both isolated from raw milk and dairy products (Stoeckel et al., 2016; Von Neubeck et al., 2016). These sequence types accounted for up to 73.87% of the cumulative similarity of these 11-day replicates.

Immediately after inoculation the rumen community composition was comprised of OTUs closely related (>97%) bacteria typically dominant in the rumen environment according to the study of Henderson et al. (2015). which looked at the rumen microbial community compositions of >700 samples from 32 animal species and 35 countries. These OTUs included *Prevotella* and *Ruminococcus*, as well as other OTUs related to *Succiniclasticum, Mollicutes*, and *Paraprevotella*, and members of the *Erysipelotrichaceae* and *Victivallaceae* families (Henderson et al., 2015). Although less specifically, *Lutispora* and *Millionella* also belong to the commonly found rumen orders *Bacteroidales* and *Clostridia*, respectively (Henderson et al., 2015). After 11 days of enrichment, a complete shift in the community was observed, which was now dominantly comprised of *Leuconostoc* OTUs (a common rumen member) and the non-prevalent, *Carnobacterium* and *Enterococcus* taxa also known to occur in the rumen. In contrast, *Pseudomonas* and *Aeromonas* are not considered part of the rumen bacterial composition, however, it must be noted that the same OTUs similar to both *L. mesenteroides* and *P. weihenstephanensis* were also enriched in at least one of the inocula blanks.

The sludge average bacterial community showed an initial composition most closely related to: *Pseudomonas caeni* (99%), isolated from the sludge of an anaerobic ammonium-oxidising bioreactor (Xiao et al., 2009); *Proteiniphilum saccharofermentans* (96%), isolated from mesophilic laboratory-scale biogas reactors (Hahnke et al., 2016); a member of the genus *Clostridium* (*Clostridium papyrosolvens*, 91% similarity isolated from the effluent of a paper-mill 42) and an OTU assigned to the phylum bacteroidetes [*Lentimicrobium saccharophilum*, 87%, novel species from a novel family isolated from a methanogenic granular sludge (Madden et al., 1989; Sun et al., 2016)]. Most *Acinetobacter* species (98% similarity) are typical members of aerobic granular sludges (Adav et al., 2009). After 11 days of enrichment only the OTU related to *P. caeni* remained as part of the most abundant OTUs in sludge inoculated microcosms which was now dominated by a *Clostridium* species (99% similarity) a commonly reported member of anerobic sludge bacterial communities (Fang et al., 2002). *Macellibacteroides fermentans* (98% similarity), an obligate anaerobe isolated from an anaerobic philtre treating wastewater from an abattoir (Jabari et al., 2012) was also enriched along with *L. mesenteroides* and *P. proteolytica* (both 99% similarity) which as mentioned above could probably have emerged from the substrate, as found in at least one of the substrate only controls and the rumen inoculated microcosms.

The most frequent and abundant OTUs accounting for about 50% of the community composition in the initial R + S community were mostly likely derived from the sludge component, i.e., OTUs related to *P. caeni, P. saccharofermentans, M. fermentans, L. saccharophilum*, and *C. papyrosolvens* along with *Leptolinea tardivalis*, which has been isolated from mesophilic anaerobic granular sludge (Yamada et al., 2006). Since the SIMPER analysis had a cut off at 70% cumulative composition, other ruminal derived bacteria might not be included in the list (Table 3). After 11 days of incubation, the mixed-source community was comprised by a balanced enrichment of bacteria also observed in the sludge (*Clostridium, P. caeni*, and *Acinetobacter*) and rumen (*Enterococcus, Carnobacterium*, and *Aeromonas*) enrichments, thus supporting the central hypothesis of this study. The initial composition and enrichment of different OTUs closely related to bacteria known to be found in the original inocula sources, rumen and sludge, validates the enrichment from these inocula despite of the growth of bacteria that might derive directly from the substrate and also observed in the mixed community, i.e., *P. weihenstephanensis* and *L. mesenteroides*. Additionally, in agreement with the evenness indices, the results for the SIMPER analysis corroborated the sludge and particularly R + S communities evenness increased with enrichment (see contribution percentages in Table 3), effect that could provide stability to these communities (Begon et al., 2007).

In summary, these results confirmed the observations from the previous two independent experiments, where rumen produced higher EtOH concentrations when inoculated at initially neutral pH, while sludge showed the best ethanologenic performance at initially acidic pH. Additionally, microcosms inoculated with a mixture 1:1 (w/w) of these inocula (R + S) significantly produced the same EtOH concentrations than the original inocula sources at both initial pH incubation conditions (pH 7 and 5.5).

## Discussion

Ethanologenic fermentation appears to be a common property of microbial communities from the diverse environments tested individually in this study, however, their performances were different in response to the imposed experimental conditions, indicating that the soluble end-products formed not only depended on the operational conditions, but also in the microbial community composition and dynamics of each system. This observation goes against a common statement in MCF experimental and modelling studies where pH has been proposed as a principal variable to direct and control the product spectrum in these systems regardless of the inoculum used (Rodríguez et al., 2006, 2008; Temudo et al., 2007). Interestingly, in this work the interaction of both initial pH and inoculum, was shown to drive in opposite directions the ethanologenic activity of rumen and sludge inocula, where maximal EtOH production was achieved at an initial neutral pH or at an initial acidic pH, respectively. Furthermore, R + S, the mixed-source community enriched from the combination of the latter inocula sources, produced EtOH at a wider pH range than the individual inocula of which it was enriched.

Important for practical applications is that (i) despite fermentation being an anaerobic process, no significant differences in EtOH production or the time taken to achieve maximal concentrations was observed when the process was initiated under aerobic conditions; and that (ii) a moderately initially acidic pH (pH 5.5) had a twofold effect: it did not negatively affect ethanol production, and promoted EtOH being the main soluble fermentation product by most of the inocula tested.

### EtOH as Major Fermentation Product at Initially Acidic pH

In contrast to the EtOH being the main soluble end-product of some single species fermentative metabolism [e.g., *S. cerevisiae*; (Madigan, 2015)], the inocula tested in this study were expected to yield EtOH at lower concentrations because of their complexity. The likely reasons are twofold: energy production in the form of ATP is low in anaerobic fermentative metabolism (as opposed to respiration) (White, 2007), and it is generally accepted that microbial populations goal is to grow (biomass production), therefore it can be assumed that under any given environmental condition, microbial metabolism will follow the most energy yielding pathways. During substrate level phosphorylation (SLP), ethanol production does not contribute to direct ATP formation, while acetic and butyric acids do (2 and 1 ATP molecules, respectively) (Rodríguez et al., 2006; White, 2007; Temudo et al., 2008).

Additionally, based on the anaerobic food chain, once secreted, EtOH could be further oxidised by some other inoculum member (e.g., acetogens) (White, 2007), eventually leading to methane and carbon dioxide as final products (Bertsch et al., 2016). Circumstance that could help explain the observations presented in the metabolic profiles of inocula under initial aerobic/neutral conditions (Figure 1A) and particularly of sludge under acidic conditions (Figure 1B), where the ethanol peak concentration is followed by a constant decline. Nonetheless, EtOH was the main soluble fermentation product in rumen inoculated microcosms at initial neutral pH, and in all the microcosms incubated under initial acidic pH and anaerobic conditions.

Other studies working with complex natural inocula degrading cellulose in batch systems, have reported EtOH (Lin et al., 2011) or the product pair EtOH/acetic acid (Ronan et al., 2013) as major fermentation products. However, these results were obtained at an initial pH range of 7–8, with a drop to pH 6 followed by a recovery to neutrality. These studies were done at temperature >50°C, reporting peak EtOH titers of 13.02 and 12.0 mM, respectively, in both cases lower to the ∼30 mM achieved here.

Although it was not possible to find similar results from comparable studies to the presented here in the literature, diverse projects have examined the effect of pH on the MCF of glucose, with the work from Temudo et al. (2007) providing a detailed carbon balance analysis of the shifts in the generation of diverse gaseous and soluble fermentation products by a mixture of two sludge sources as inoculum growing in a chemostat as a function of pH. However, the results from this study indicated pH higher to 6.5 would favour acetate/EtOH production.

Furthermore, based on the latter study, González-Cabaleiro et al. (2015) constructed a metabolic model which largely agreed with those experimental observations, apart from the discrepancy of whether propionate or formate accompany acetate production at high pH values in the switch from butyrate formation, this model predicted EtOH production to be favoured at high pH, particularly at the 8–8.5 pH range.

Contrarily, using the model of Rodríguez et al. (2006) as reference, EtOH production would be favoured at environmental pH below 5.6, as a low pH would make excretion of acids against the chemical gradient. Additionally, although the ionised form of these products could then be excreted, the cell membrane negatively charged, forces the movement of ionised molecules to be actively mediated (González-Cabaleiro et al., 2015), exceeding the energy benefits of acetic or butyric acid production. Moreover, the bacterial production of EtOH typically involves the oxidation of 2 molecules of NADH, thus regenerating NAD^+^, a fundamental electron carrier for metabolic redox reactions, including ATP synthesis, ATP hydrolysis and ultimately biomass production.

In agreement with these predictions, almost all the microcosms incubated with initial acidic pH (∼5.3) had EtOH as the major soluble end-product. The exception being rumen under initial aerobic conditions, which showed a high, but variable, butanol production.

Another experimental observation that can be linked to external pH driving fermentation products formation as predicted by Rodríguez et al. (2006), is the dominance of butyric acid closely followed by acetic acid in microcosms incubated at initial neutral pH. As the pH of the media moved from ∼7 to ∼5.5, acetic acid decreased likely due to the active transport energy cost associated with the movement of its ionised form across the membrane, yet butyric acid production, although less efficient in ATP formation than acetic acid, mediates the synthesis of ATP and NAD^+^ regeneration (White, 2007).

Interestingly, rumen was the exception in this case, producing EtOH as major fermentation product when starting at pH 6.9 and up to a minimum of pH 5.7, theoretically still favourable for butyrate formation. Thus agreeing with the predictions of Temudo et al. (2007) and González-Cabaleiro et al. (2015).

Although in conjunction, the three studies previously described can provide an overall explanation of what has been experimentally observed, it must be noted that they solely considered the fermentation of glucose as carbon source at 30°C under steady state conditions achieved by means of a chemostat type reactor operation or simulation. In addition, the metabolic models were constructed treating the microbial community as a single organism, neglecting phylogenetic diversity and assuming the environmental conditions direct product formation based on the thermodynamics of maximum energy yield. While the cause for the disagreement between these reports regarding pH and soluble fermentation products formation is not entirely clear, González-Cabaleiro et al. (2015) coupled FADH_2_ to the reduction of acetaldehyde to ethanol, which in their words, was necessary to ensure the prediction of the observed experimentally by Temudo et al. (2007), but otherwise, a mechanism without foundation in the literature. On the other hand, Rodríguez et al. (2006) only accounted for NADH/NAD^+^ in their model, which could be an over simplification. Also, the works from Temudo et al. (2007) and González-Cabaleiro et al. (2015) operated under substrate limitation (4 g/L) of glucose, whereas Rodríguez et al. (2006) utilised a higher concentration of this sugar (10 g/L), and it has been observed that substrate concentration is another environmental factor affecting MCF product spectrum (Rodríguez et al., 2008; Temudo et al., 2008). In the present project, the concentration of OMSW was 50 g/L, likely causing further deviation from the glucose-based studies.

The metabolic energetics-based models proved to be useful tools to explain the general patterns observed experimentally, however, one of the most important limitations of their predictions is the neglection of community composition, thus ignoring the potential existence of microbial interactions that would make possible otherwise thermodynamically unfavourable reactions. Moreover, glucose was considered the only carbon source, which is a valid generalisation as the hydrolysis of cellulose, starch and other simpler carbohydrates would follow glucose metabolism, but under represents the complex composition of OMSW.

The disagreement of the predictions and the lack of further information in the literature regarding direct EtOH production by rumen as inoculum impose a limitation in the explanation of the results observed.

### Enrichment of a Mixed-Source Novel Community

Nevertheless, EtOH production profiles along with 16S RNA sequencing analyses demonstrated the successful enrichment of a novel mixed-source community, whereby bacteria derived from both original inocula seemed to equally contribute to the total relative abundance, with the additional enrichment of bacteria probably derived from the substrate itself.

Several studies have used a number of sludge sources as inocula for mixed culture fermentation (MCF) (Fang et al., 2002; Wongwilaiwalin et al., 2010; Liang et al., 2014; Liu et al., 2016) while the utilisation of rumen in this type of systems has not been reported. Prior work using sludge, manure, compost and soil as inocula has demonstrated changes in microbial community composition in relation to pH (Temudo et al., 2008; Lauber et al., 2009; Karadag and Puhakka, 2010), however, the comparison of communities arising from different inocula sources has been sparingly explored (Peacock et al., 2013; Jimenez et al., 2014; Simmons et al., 2014). Even more, although some studies have mixed different inocula sources for the inoculation of MCF reactors, the determination of whether the enriched community and its function was derived from the combination of this sources has been neglected. This could be due to the usual enrichment approaches used employing sequential transfers until stable degradation/fermentation activity is reached in batch reactors (Haruta et al., 2002; Kato et al., 2004; Guo et al., 2010; Ronan et al., 2013) or until steady state conditions are established in continuous reactors (Temudo et al., 2008; Sun et al., 2015; Wu et al., 2017). In these studies, the bacterial community compositions were only determined at this stage for potential isolation of the most abundant members (Kato et al., 2005; Wahyudi et al., 2010; Zhou et al., 2015), or for the general characterisation of the system (Temudo et al., 2008; Lin et al., 2011; Ronan et al., 2013). Although this is understandable, since the final community would be the one used for further production, elucidating the mechanisms of enrichment, adaptation and potential enhanced capabilities could also provide more efficient identification of promising communities/species for the transformation of specific lignocellulosic substrates into the desired metabolic products (Garcia et al., 2011; Peacock et al., 2013; Jimenez et al., 2014). Additionally, the initial study of community composition is particularly relevant for mixed-source inocula projects, since the combination of inocula may not even be necessary, thus simplifying the process for future reproducibility.

Here, although only the R + S community enriched at an initial pH of 5.5 was analysed, functional redundancy, resulting from the mixing process, can be assumed because of: (i) the presence of both ruminal and sludge derived OTUs in the enriched community; and (iii) the fact that EtOH production by this community was not significantly different to that of the rumen when inoculated at an initial pH of 7 or the sludge at an initial pH of 5.5.

### Putative Functions of the Enriched Communities’ Members

Rumen and anaerobic granular sludge are specialised anaerobic environments where organic matter fermentation is a major community function (Schmidt and Ahring, 1996; Flint, 1997). However, inherent differences in their natural microbial composition and commonly degraded carbon sources were expected to select for different bacteria in the OMSW enrichments. Additionally, since the pH of these inocula sources before incubation was in both cases close to neutrality (7.14 ± 0.02, 6.91 ± 0.02, rumen and sludge, respectively), their opposite behaviour regarding EtOH production over the initial pH range tested is not directly attributable to pH selection at source, but unknown factors which have controlled the composition of their respective communities.

Broadly, it can be inferred that all the enriched communities (considering OTUs contributing to the 70% of the total relative abundance) displayed linked trophic interactions, whereby putatively aerobic (*Pseudomonas* and *Aeromonas*) and facultative anaerobes (some *Bacilli* members) might have initiated the OMSW consumption of the readily available sugars dissolved, causing CO_2_ accumulation and O_2_ consumption, thus providing an appropriate environment for the growth of obligate anaerobes (*Clostridia* and some *Bacilli* members) capable of fermentation and degradation of more complex organic matter (cellulose). This degradation likely releases simpler organic compounds into the media, as previously proposed in other studies of MCF initially incubated under aerobic conditions degrading and fermenting cellulosic substrates (Kato et al., 2005; Guo et al., 2010).

However, to elucidate how this seemingly straight forward mechanism led to different ethanologenic activity by the inocula tested here in response to the initial pH of the media, a closer inspection to the OTUs found to be typical in each enrichment (Table 3) was conducted and compared against the literature.

**FIGURE 5:**
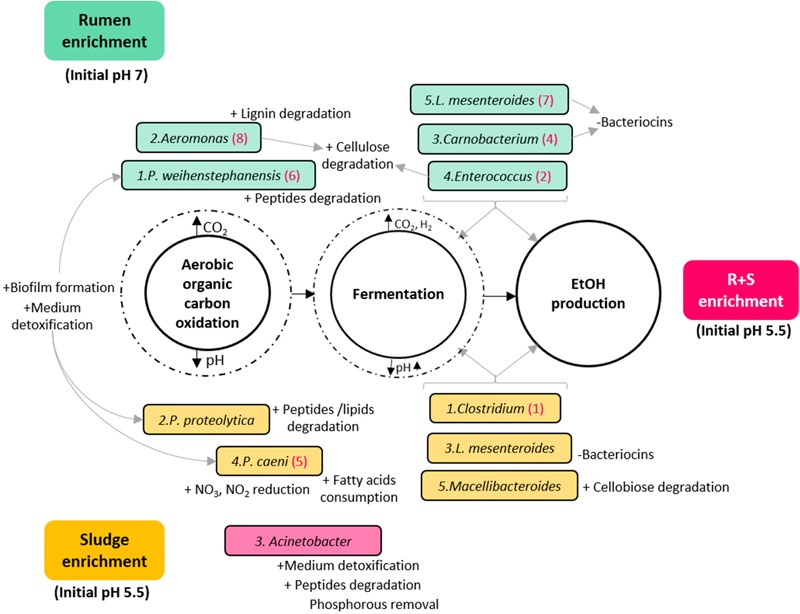
Diagram of the putative functions of the dominant OTUs enriched from the different inocula under study. Turquoise rectangles: rumen enriched. Yellow rectangles: sludge enriched. Pink rectangle: R + S enriched. The numbers before the species name indicate in hierarchical order their contribution to the total relative abundance of their community. Numbers in brackets after the species name, signal OTUs also enriched in the R + S community in hierarchical order of contribution to the total relative abundance for this community. ± symbols before specific OTUs putative functions indicate an activity possibly benefiting (+) or damaging (-) the community.

Based on the general metabolic activities of the dominant OTUs, the MCF process could be simply divided in two major metabolic functions: organic carbon oxidation under aerobic conditions, followed by fermentation by facultative anaerobes/obligate anaerobes once most of the oxygen had been consumed with concomitant carbon dioxide accumulation and pH drop (Figure 5). Since this work is focused on EtOH production, a third metabolic distinction could be proposed between general fermenters and those with potentially higher EtOH production (numbered in hierarchical order for each community under the assumption of higher metabolic activity by the most abundant organism).

As expected, functional redundancy occurred not only between but within communities, and it was not exclusive of the general divisions among aerobic oxidation and fermentation of organic matter, but also of the particular traits that could benefit or damage the communities. It is worth highlighting here that the abundant enrichment of bacteria able to degrade aromatic compounds and detoxify the medium may indicate that the acid pre-treatment without further conditioning imposed an additional constraint for the enrichment.

Another important finding is the co-enrichment of rumen and sludge-derived OTUs in the R + S community. As these OTUs were not present or highly abundant directly after inoculation in the R + S microcosms, it is safe to assume their actual enrichment, which provided the R + S community with further functional redundancy and indicates *Clostridium* and *Enterococcus* might be the major EtOH producers in the original inocula enrichments. This result also demonstrated that most of the OTU enriched were capable of function and even to produce EtOH under acidic conditions (pH > 4.5), questioning which mechanisms occurring in R + S allowed the enrichment of ruminal-derived bacteria under initial pH 5.5, when it was previously shown (Figure 3) the low ethanol production by this inocula when incubated at initial acidic pH. Future work should look at how these communities can be optimised and maintained.

## Conclusion

It was demonstrated that although initial pH can be considered a key environmental factor for ethanol production from the organic fraction of municipal solid waste, the inoculum selection also has a significant effect in the formation of this soluble end-product, as the interaction of both factors was shown to drive a divergence in the ethanologenic activity of rumen and sludge inocula, where maximal EtOH production was achieved at an initial neutral pH or at an initial acidic pH, respectively. The mixed-source community proved to be enriched from the combination of these inocula and produced EtOH under both initial pH conditions, likely due to its high functional redundancy. At the moment of writing, this work is the first determining whether the initial ethanologenic mixed-source community was indeed comprised of members belonging to the original inocula sources. This information is relevant when different inocula are combined for MCF studies, as it experimentally demonstrates the benefits of diversity and function assembled from different sources. Besides, since the combination of inocula could not be even necessary, this information could help to simplify batch processes inoculation.

## Ethics Statement

The Ethical review of this study was conducted by the Policy & Information Team, Newcastle University. This “low risk” study did not involve animal subjects, human subjects/data nor clinical trials.

## Author Contributions

PC-B conceived and designed the study, performed the experiments, analysed and interpreted the data, and drafted the manuscript. BB importantly contributed to the data acquisition and interpretation of GC-FID measurements. NG guided the experiments and analysis of the data and was a major contributor in the review of the manuscript. JD and PS were involved in revising the manuscript for important intellectual content. All authors read and approved the final manuscript.

## Conflict of Interest Statement

The authors declare that the research was conducted in the absence of any commercial or financial relationships that could be construed as a potential conflict of interest.

## References

[B1] Adav S. S. Lee D. J. Lai J. Y. (2009). Proteolytic activity in stored aerobic granular sludge and structural integrity. *Bioresour. Technol.* 100 68–73. 10.1016/j.biortech.2008.05.045 18614356

[B2] Agrawal R. Satlewal A. Gaur R. Mathur A. Kumar R. Gupta R. P. (2015). Pilot scale pretreatment of wheat straw and comparative evaluation of commercial enzyme preparations for biomass saccharification and fermentation. *Biochem. Eng. J.* 102 54–61. 10.1016/j.bej.2015.02.018

[B3] APHA (2012). *2540 G. Total, Fixed, and Volatile Solids in Solid and Semisolid Samples. in STANDARD Methods for the Examination of Water and Wastewater.* Washington, D.C: APHA.

[B4] Balat M. (2011). Production of bioethanol from lignocellulosic materials via the biochemical pathway: a review. *Energy Convers. Manag.* 52 858–875. 10.1016/j.enconman.2010.08.013

[B5] Bayard R. Benbelkacem H. Gourdon R. Buffière P. (2018). Characterization of selected municipal solid waste components to estimate their biodegradability. *J. Environ. Manag.* 216 4–12. 10.1016/j.jenvman.2017.04.087 28506668

[B6] Begon M. Colin R. T. Harper J. L. (2007). “Ecology – From Individuals to Ecosystems,” in *Biological Conservation* 4th Edn Vol. 135 eds Begon M. Townsend C. R. Harper J. L. (Malden, MA: Blackwell Publishing) 10.1016/j.biocon.2006.10.034

[B7] Bertsch J. Siemund A. L. Kremp F. Müller V. Wolfgang J. Str M. (2016). A novel route for ethanol oxidation in the acetogenic bacterium Acetobacterium woodii: the acetaldehyde / ethanol dehydrogenase pathway. *Environ. Microbiol.* 18 2913–2922. 10.1111/1462-2920.13082 26472176

[B8] Blake L. I. Halim F. A. Gray C. Mair R. Manning D. A. C. Sallis P. (2017). Evaluating an anaerobic digestion (AD) feedstock derived from a novel non-source segregated municipal solid waste (MSW) product. *Waste Manag.* 59 149–159. 10.1016/j.wasman.2016.10.031 27818071

[B9] Boratyn G. M. Schäffer A. A. Agarwala R. Altschul S. F. Lipman D. J. Madden T. L. (2012). Domain enhanced lookup time accelerated BLAST. *Biol. Dir.* 7:12. 10.1186/1745-6150-7-12 22510480 PMC3438057

[B10] Bozorgirad M. A. Zhang H. Haapala K. R. Murthy G. S. (2013). Environmental impact and cost assessment of incineration and ethanol production as municipal solid waste management strategies. *Int. J. Life Cycle Assess.* 18 1502–1512. 10.1007/s11367-013-0587-z

[B11] Carrillo-Barragan P. (2018). Data from: enrichment and characterisation of a mixed-source ethanologenic community degrading the organic fraction of municipal solid waste under minimal environmental control. *Zenodo Digit. Repository* 1 1–8. 10.5281/zenodo.2098592 PMC6465759 31024500

[B12] Cheng J. R. Zhu M. J. (2012). A novel co-culture strategy for lignocellulosic bioenergy production: a systematic review. *Int. J. Mod. Biol. Med.* 1 166–193.

[B13] Clarke K. R. Gorley R. N. (2015). *PRIMER v7: User Manual/Tutorial.* Plymouth: PRIMER-E Ltd.

[B14] D’Amore R. Ijaz U. Z. Schirmer M. Kenny J. G. Gregory R. Darby A. C. (2016). A comprehensive benchmarking study of protocols and sequencing platforms for 16S rRNA community profiling. *BMC Genomics* 17:55. 10.1186/s12864-015-2194-9 26763898 PMC4712552

[B15] De Wit R. Bouvier T. (2006). *‘Everything is everywhere*, but, *the environment selects*’; what did Baas Becking and Beijerinck really say?. *Environ. Microbiol.* 8 755–758. 10.1111/j.1462-2920.2006.01017.x 16584487

[B16] Du R. Yan J. Li S. Zhang L. Zhang S. Li J. (2015). Cellulosic ethanol production by natural bacterial consortia is enhanced by pseudoxanthomonas taiwanensis. *Biotechnol. Biofuels* 8 1–10. 10.1186/s13068-014-0186-7 25648981 PMC4308921

[B17] Durán-Moreno A. Garcés M. Velasco A. Marín J. Gutiérrez R. Moreno A. (2013). Mexico City’s municipal solid waste characteristics and composition analysis. *Revista Internacional de Contaminación Ambiental* 29 39–46.

[B18] Fang H. H. P. Zhang T. Liu H. (2002). Microbial diversity of a mesophilic hydrogen-producing sludge. *Appl. Microbiol. Biotechnol.* 58 112–118. 10.1007/s00253-001-0865-8 11833529

[B19] Flint H. J. (1997). The rumen microbial ecosystem–some recent developments. *Trends Microbiol.* 5 483–488. 10.1016/S0966-842X(97)01159-1 9447660

[B20] Frankó B. Galbe M. Wallberg O. (2016). Bioethanol production from forestry residues: a comparative techno-economic analysis. *Appl. Energy* 184 727–736. 10.1016/j.apenergy.2016.11.011

[B21] Ganigué R. Sánchez-paredes P. Bañeras L. Colprim J. (2016). Low fermentation pH Is a trigger to alcohol production, but a killer to chain elongation. *Front. Microbiol.* 7:702. 10.3389/fmicb.2016.00702 27252682 PMC4877396

[B22] Garcia S. L. Jangid K. Whitman W. B. Das K. C. (2011). Transition of microbial communities during the adaption to anaerobic digestion of carrot waste. *Bioresour. Technol.* 102 7249–7256. 10.1016/j.biortech.2011.04.098 21620691

[B23] González-Cabaleiro R. Lema J. M. Rodríguez J. (2015). Metabolic energy-based modelling explains product yielding in anaerobic mixed culture fermentations. *PLoS One* 10:e0126739. 10.1371/journal.pone.0126739 25992959 PMC4436308

[B24] Guo P. Zhu W. Wang H. Lü Y. Wang X. Zheng D. (2010). Functional characteristics and diversity of a novel lignocelluloses degrading composite microbial system with high xylanase activity. *J. Microbiol. Biotechnol.* 20 254–264. 10.4014/jmb.0906.06035 20208427

[B25] Hahnke S. Langer T. Koeck D. E. Klocke M. (2016). Description of proteiniphilum saccharofermentans sp. nov., petrimonas mucosa sp. nov. and *Fermentimonas caenicola* gen. nov., sp. nov., isolated from mesophilic laboratory-scale biogas reactors, and emended description of the genus Proteiniphilum. *Int. J. Syst. Evol. Microbiol.* 66 1466–1475. 10.1099/ijsem.0.000902 26782749

[B26] Hames B. Ruiz R. Scarlata C. Sluiter A. Sluiter J. Templeton D. (2008). *Preparation of Samples for Compositional Analysis Laboratory Analytical Procedure (LAP).* Technical Report NREL/TP-510-42620. Golden, CO: National Renewable Energy Laboratory.

[B27] Haruta S. Cui Z. Huang Z. Li M. Ishii M. Igarashi Y. (2002). Construction of a stable microbial community with high cellulose-degradation ability. *Appl. Microbiol. Biotechnol.* 59 529–534. 10.1007/s00253-002-1026-1024 12172621

[B28] Henderson G. Cox F. Ganesh S. Jonker A. Young W. Janssen P. H. (2015). Rumen microbial community composition varies with diet and host, but a core microbiome is found across a wide geographical range. *Sci. Rep.* 5 1–13. 10.1038/srep14567 26449758 PMC4598811

[B29] Hong K. K. Nielsen J. (2012). Metabolic engineering of Saccharomyces cerevisiae: a key cell factory platform for future biorefineries. *Cell. Mol. Life Sci.* 69 2671–2690. 10.1007/s00018-012-0945-1 22388689 PMC11115109

[B30] Jabari L. Gannoun H. Cayol J. L. Hedi A. Sakamoto M. Falsen E. (2012). Macellibacteroides fermentans gen. nov., sp. nov., a member of the family Porphyromonadaceae isolated from an upflow anaerobic filter treating abattoir wastewaters. *Int. J. Syst. Evol. Microbiol.* 62 2522–2527. 10.1099/ijs.0.032508-0 22180609

[B31] Jimenez D. J. Dini-Andreote F. Van Elsas J. D. (2014). Metataxonomic profiling and prediction of functional behaviour of wheat straw degrading microbial consortia. *Biotechnol. Biofuels* 7:92. 10.1186/1754-6834-7-92 24955113 PMC4064818

[B32] Jönsson L. J. Alriksson B. Nilvebrant N. O. (2013). Bioconversion of lignocellulose: inhibitors and detoxification. *Biotechnol. Biofuels* 6 1–10. 10.1186/1754-6834-6-16 23356676 PMC3574029

[B33] Jönsson L. J. Martín C. (2016). Pretreatment of lignocellulose: formation of inhibitory by-products and strategies for minimizing their effects. *Bioresour. Technol.* 199 103–112. 10.1016/j.biortech.2015.10.009 26482946

[B34] Kádár Z. Maltha S. F. Szengyel Z. Réczey K. De.Laat W. (2007). Ethanol fermentation of various pretreated and hydrolyzed substrates at low initial pH. *Appl. Biochem. Biotechnol.* 137–140 847–858. 10.1007/s12010-007-9102-y 18478439

[B35] Kalogo Y. Habibi S. Maclean H. L. Joshi S. V. (2007). Environmental implications of municipal solid waste-derived ethanol. *Environ. Sci. Technol.* 41 35–41. 10.1021/es061117b 17265924

[B36] Karadag D. Puhakka J. A. (2010). Direction of glucose fermentation towards hydrogen or ethanol production through on-line pH control. *Int. J. Hydrogen Energy* 35 10245–10251. 10.1016/j.ijhydene.2010.07.139

[B37] Kato S. Haruta S. Cui Z. J. Ishii M. Igarashi Y. (2004). Effective cellulose degradation by a mixed-culture system composed of a cellulolytic clostridium and aerobic non-cellulolytic bacteria. *FEMS Microbiol. Ecol.* 51 133–142. 10.1016/j.femsec.2004.07.015 16329862

[B38] Kato S. Haruta S. Cui Z. J. Ishii M. Igarashi Y. (2005). Stable coexistence of five bacterial strains as a cellulose-degrading community. *Appl. Environ. Microbiol.* 71 7099–7106. 10.1128/AEM.71.11.7099-7106.2005 16269746 PMC1287685

[B39] Kleerebezem R. van Loosdrecht M. C. (2007). Mixed culture biotechnology for bioenergy production. *Curr. Opin. Biotechnol.* 18 207–212. 10.1016/j.copbio.2007.05.001 17509864

[B40] Lampert Y. Dror B. Sela N. Teper-Bamnolker P. Daus A. Sela Saldinger S. (2017). Emergence of *Leuconostoc mesenteroides* as a causative agent of oozing in carrots stored under non-ventilated conditions. *Microb. Biotechnol.* 10 1677–1689. 10.1111/1751-7915.12753 28834204 PMC5658626

[B41] Lauber C. L. Hamady M. Knight R. Fierer N. (2009). Pyrosequencing-based assessment of soil pH as a predictor of soil bacterial community structure at the continental scale. *Appl. Environ. Microbiol.* 75 5111–5120. 10.1128/AEM.00335-09 19502440 PMC2725504

[B42] Leme M. M. V. Rocha M. H. Lora E. E. S. Venturini O. J. Lopes B. M. Ferreira C. H. (2014). Techno-economic analysis and environmental impact assessment of energy recovery from municipal solid waste (MSW) in Brazil. *Resour. Conserv. Recycl.* 87 8–20. 10.1016/j.resconrec.2014.03.003

[B43] Li A. Antizar-Ladislao B. Khraisheh M. (2007). Bioconversion of municipal solid waste to glucose for bio-ethanol production. *Bioprocess Biosyst. Eng.* 30 189–196. 10.1007/s00449-007-0114-3 17458580

[B44] Li S. Zhang X. Andresen J. M. (2012). Production of fermentable sugars from enzymatic hydrolysis of pretreated municipal solid waste after autoclave process. *Fuel* 92 84–88. 10.1016/j.fuel.2011.07.012

[B45] Liang S. McDonald A. G. Coats E. R. (2014). Lactic acid production with undefined mixed culture fermentation of potato peel waste. *Waste Manag.* 34 2022–2027. 10.1016/j.wasman.2014.07.009 25127412

[B46] Life Technologies (2012). *Ion Amplicon Library Preparation (Fusion Method) for Use With: Ion Torrent Personal Genome Machine^®^ System.* Carlsbad, CA: Life Technologies.

[B47] Lin C. W. Wu C. H. Tran D. T. Shih M. C. Li W. H. Wu C. F. (2011). Mixed culture fermentation from lignocellulosic materials using thermophilic lignocellulose-degrading anaerobes. *Process Biochem.* 46 489–493. 10.1016/j.procbio.2010.09.024

[B48] Liu J. H. Zhang M. L. Zhang R. Y. Zhu W. Y. Mao S. Y. (2016). Comparative studies of the composition of bacterial microbiota associated with the ruminal content, ruminal epithelium and in the faeces of lactating dairy cows. *Microb. Biotechnol.* 9 257–268. 10.1111/1751-7915.12345 26833450 PMC4767291

[B49] Madden R. H. Bryder M. J. Poole N. J. (1989). Isolation and characterization of an anaerobic, cellulolytic bacterium, clostridium papyrosolvens sp. nov. *Int. J. Syst. Bacteriol.* 39 68–71. 10.1099/00207713-39-1-68

[B50] Madigan M. (2015). *Brock Biology of Microorganisms* 14 Edn. San Francisco, CA: Pearson Education.

[B51] Mohd Azhar S. H. Abdulla R. Jambo S. A. Marbawi H. Gansau J. A. Mohd Faik A. A. (2017). Yeasts in sustainable bioethanol production: a review. *Biochem. Biophys. Rep.* 10 52–61. 10.1016/j.bbrep.2017.03.003 29114570 PMC5637245

[B52] Okeke B. C. Lu J. (2011). Characterization of a defined cellulolytic and xylanolytic bacterial consortium for bioprocessing of cellulose and hemicelluloses. *Appl. Biochem. Biotechnol.* 163 869–881. 10.1007/s12010-010-9091-0 20859703

[B53] Oksanen J. Guillaume Blanchet F. Friendly M. Kindt R. Legendre P. McGlinn D. (2018). *Vegan: Community Ecology Package. R Package Version 2.4-6*. Available at: https://CRAN.R-project.org/package=vegan (accessed June 06, 2018).

[B54] Peacock J. P. Cole J. K. Murugapiran S. K. Dodsworth J. A. Fisher J. C. Moser D. P. (2013). Pyrosequencing reveals high-temperature cellulolytic microbial consortia in great boiling spring after in situ lignocellulose enrichment. *PLoS One* 8:e59927. 10.1371/journal.pone.0059927 23555835 PMC3612082

[B55] Quast C. Pruesse E. Yilmaz P. Gerken J. Schweer T. Yarza P. (2013). The SILVA ribosomal RNA gene database project: improved data processing and web-based tools. *Nucleic Acids Res.* 41 D590–D596. 10.1093/nar/gks1219 23193283 PMC3531112

[B56] Quested T. Johnson H. (2009). *Household Food and Drink Waste in the UK: A Report Containing Quantification of the Amount and Types of Household Food and Drink Waste in the UK.* Banbury: Waste and Resources Action Programme.

[B57] Quince C. Lanzen A. Davenport R. J. Turnbaugh P. J. (2011). Removing noise from pyrosequenced amplicons. *BMC Bioinformatics* 12:38. 10.1186/1471-2105-12-38 21276213 PMC3045300

[B58] Rice E. W. Baird R. B. Eaton A. D. Clesceri L. S. (2012). *Standard Methods for the Examination of Water and Wastewater* 22nd Edn. New York, NY: American Public Health Association.

[B59] Rittmann B. E. McCarty P. L. (2001). *Environmental Biotechnology: Principles and Applications.* Boston, MA: McGraw-Hill 10.1016/S0958-1669(96)80047-4

[B60] Rodríguez J. Kleerebezem R. Lema J. M. Van Loosdrecht M. C. M. (2006). Modeling product formation in anaerobic mixed culture fermentations. *Biotechnol. Bioeng.* 93 592–606. 10.1002/bit.20765 16273553

[B61] Rodríguez J. Lema J. M. Kleerebezem R. (2008). Energy-based models for environmental biotechnology. *Trends Biotechnol.* 26 366–374. 10.1016/j.tibtech.2008.04.003 18513813

[B62] Ronan P. Yeung C. W. Schellenberg J. Sparling R. Wolfaardt G. M. Hausner M. (2013). A versatile and robust aerotolerant microbial community capable of cellulosic ethanol production. *Bioresour. Technol.* 129 156–163. 10.1016/j.biortech.2012.10.164 23238345

[B63] Sarkar N. Ghosh S. K. Bannerjee S. Aikat K. (2012). Bioethanol production from agricultural wastes: an overview. *Renew. Energy* 37 19–27. 10.1016/j.renene.2011.06.045 20662378

[B64] Schloss P. D. Gevers D. Westcott S. L. (2011). Reducing the effects of PCR amplification and sequencing artifacts on 16s rRNA-based studies. *PLoS One* 6:e27310. 10.1371/journal.pone.0027310 22194782 PMC3237409

[B65] Schloss P. D. Westcott S. L. Ryabin T. Hall J. R. Hartmann M. Hollister E. B. (2009). Introducing mothur: open-source, platform-independent, community-supported software for describing and comparing microbial communities. *Appl. Environ. Microbiol.* 75 7537–7541. 10.1128/AEM.01541-09 19801464 PMC2786419

[B66] Schmidt J. E. Ahring B. K. (1996). Granular sludge formation in upflow anaerobic sludge blanket (UASB) reactors. *Biotechnol. Bioeng.* 49 229–246. 10.1002/(SICI)1097-0290(19960205)49:3<229::AID-BIT1>3.0.CO;2-M 18623574

[B67] Simmons C. W. Reddy A. P. Simmons B. A. Singer S. W. Vandergheynst J. S. (2014). Effect of inoculum source on the enrichment of microbial communities on two lignocellulosic bioenergy crops under thermophilic and high-solids conditions. *J. Appl. Microbiol.* 117 1025–1034. 10.1111/jam.12609 25066414

[B68] Sluiter A. Hames B. Hyman D. Payne C. Ruiz R. Scarlata C. (2008). *Determination of Total Solids in Biomass and Total Dissolved Solids in Liquid Process Samples.* Technical Report NREL/TP-510-42621. Golden, CO: National Renewable Energy Laboratory.

[B69] Stoeckel M. Lidolt M. Achberger V. Glück C. Krewinkel M. Stressler T. (2016). Growth of pseudomonas weihenstephanensis, *Pseudomonas proteolytica* and Pseudomonas sp. in raw milk: impact of residual heat-stable enzyme activity on stability of UHT milk during shelf-life. *Int. Dairy J.* 59 20–28. 10.1016/j.idairyj.2016.02.045

[B70] Sun L. Pope P. B. Eijsink V. G. H. Schnürer A. (2015). Characterization of microbial community structure during continuous anaerobic digestion of straw and cow manure. *Microb. Biotechnol.* 8 815–827. 10.1111/1751-7915.12298 26152665 PMC4554469

[B71] Sun L. Toyonaga M. Ohashi A. Tourlousse D. M. Matsuura N. Meng X. Y. (2016). Lentimicrobium saccharophilum gen. nov., sp. nov., a strictly anaerobic bacterium representing a new family in the phylum bacteroidetes, and proposal of lentimicrobiaceae fam. nov. *Int. J. Syst. Evol. Microbiol.* 66 2635–2642. 10.1099/ijsem.0.001103 27098854

[B72] Sun Y. Cheng J. (2002). Hydrolysis of lignocellulosic materials for ethanol production: a review q. *Bioresour. Technol.* 83 1–11. 10.1016/S0960-8524(01)00212-7 12058826

[B73] Talebnia F. Karakashev D. Angelidaki I. (2010). Production of bioethanol from wheat straw: an overview on pretreatment, hydrolysis and fermentation. *Bioresour. Technol.* 101 4744–4753. 10.1016/j.biortech.2009.11.080 20031394

[B74] Temudo M. F. Kleerebezem R. Van Loosdrecht M. (2007). Influence of the pH on (Open) mixed culture fermentation of glucose: a chemostat study. *Biotechnol. Bioeng.* 98 69–79. 10.1002/bit.21412 17657773

[B75] Temudo M. F. Muyzer G. Kleerebezem R. Van Loosdrecht M. C. M. (2008). Diversity of microbial communities in open mixed culture fermentations: impact of the pH and carbon source. *Appl. Microbiol. Biotechnol.* 80 1121–1130. 10.1007/s00253-008-1669-x 18800185 PMC7419374

[B76] Varrone C. Heggeset T. M. B. Le S. B. Haugen T. Markussen S. Skiadas I. V. (2015). Comparison of different strategies for selection/adaptation of mixed microbial cultures able to ferment crude glycerol derived from second-generation biodiesel. *BioMed. Res. Int.* 2015:932934. 10.1155/2015/932934 26509171 PMC4609794

[B77] Von Neubeck M. Huptas C. GlÜck C. Krewinkel M. Stoecke M. Stressler T. (2016). Pseudomonas helleri sp. nov. and *Pseudomonas weihenstephanensis* sp. nov., isolated from raw cow’s milk. *Int. J. Syst. Evol. Microbiol.* 66 1163–1173. 10.1099/ijsem.0.000852 26675012

[B78] Wahyudi A. Cahyanto M. N. Soejono M. Bachruddin Z. (2010). Potency of lignocellulose degrading bacteria isolated from buffalo and horse gastrointestinal tract and elephant dung for feed fiber degradation. *J. Indonesian Trop. Anim. Agric.* 35 34–41. 10.14710/jitaa.35.1.34-41

[B79] White D. (2007). “Fermentations,” in *The Physiology and Biochemistry of Procaryotes* Third edit Edn eds White D. Drummond J. T. Fuqua C. (New York, NY: Oxford University Press) 383–403.

[B80] Wongwilaiwalin S. Rattanachomsri U. Laothanachareon T. Eurwilaichitr L. Igarashi Y. Champreda V. (2010). Analysis of a thermophilic lignocellulose degrading microbial consortium and multi-species lignocellulolytic enzyme system. *Enzyme Microb. Technol.* 47 283–290. 10.1016/j.enzmictec.2010.07.013

[B81] Wu Y. Wang C. Zheng M. Zuo J. Wu J. Wang K. (2017). Effect of pH on ethanol-type acidogenic fermentation of fruit and vegetable waste. *Waste Manag.* 60 158–163. 10.1016/j.wasman.2016.09.033 27707543

[B82] Xiao Y. P. Hui W. Wang Q. Roh S. W. Shi X. Q. Shi J. H. (2009). *Pseudomonas* caeni sp. nov., a denitrifying bacterium isolated from the sludge of an anaerobic ammonium-oxidizing bioreactor. *Int. J. Syst. Evol. Microbiol.* 59 2594–2598. 10.1099/ijs.0.005108-0 19622647

[B83] Yamada T. Sekiguchi Y. Hanada S. Imachi H. Ohashi A. Harada H. (2006). Anaerolinea thermolimosa sp. nov., *Levilinea saccharolytica* gen. nov., sp. nov. and *Leptolinea tardivitalis* gen. nov., sp. nov., novel filamentous anaerobes, and description of the new classes *Anaerolineae classis* nov. and *Caldilineae classis* nov. in the. *Int. J. Syst. Evol. Microbiol.* 56 1331–1340. 10.1099/ijs.0.64169-0 16738111

[B84] Zhang F. Zhang Y. Chen M. Zeng R. J. (2012). Hydrogen supersaturation in thermophilic mixed culture fermentation. *Int. J. Hydrogen Energy* 37 17809–17816. 10.1016/j.ijhydene.2012.09.019

[B85] Zhou K. Qiao K. Edgar S. Stephanopoulos G. (2015). Distributing a metabolic pathway among a microbial consortium enhances production of natural products. *Nat. Biotechnol.* 33 377–383. 10.1038/nbt.3095 25558867 PMC4867547

[B86] Zhou Y. J. Kerkhoven E. J. Nielsen J. (2018). Barriers and opportunities in bio-based production of hydrocarbons. *Nat. Energy* 3 925–935. 10.1038/s41560-018-0197-x

