# Supplementary material for: Enrichment and Characterisation of a Mixed-Source Ethanologenic Community Degrading the Organic Fraction of Municipal Solid Waste Under Minimal Environmental Control
Source: Front Microbiol. 2019 Apr 9;10:722. doi: 10.3389/fmicb.2019.00722 (PMC6465759; doi:10.3389/fmicb.2019.00722)
Supplement: Supplementary file 1 [file Data_Sheet_1.docx]

Supplementary Material

# Experimental designs diagrams


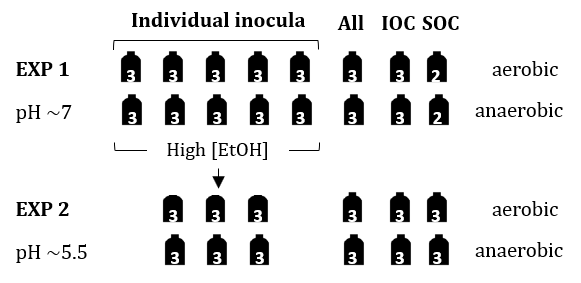
Figure S1 Diagram of the experimental design to test the effects of inocula source, oxygen presence and pH in EtOH production. Numbers inside the bottle icons indicate the number of replicates for each treatment.


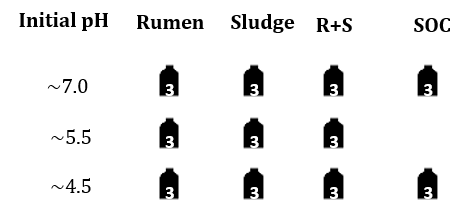
Figure S2 Diagram of the experimental design to test the effect of pH in the EtOH production of rumen, sludge and the mix of both (R+S).

# Organic Municipal Solid Waste (OMSW) analogue composition

Table S1 Composition of the OMSW analogue prepared to be used as substrate for EtOH production in this study.

| MSW organic fraction category | | %  OMSW analogue |
| --- | --- | --- |
| Food waste  (Organics) |  |  |
|  | Vegetables (spring greens) | 31.3 |
|  | Fruits (apple cores; banana, oranges and watermelon peelings) | 17.6 |
|  | Bakery (baguette) | 13.8 |
|  | Meals (fried rice) | 10.8 |
|  | Meat (beef meatballs) | 9.5 |
|  |  |  |
| Paper | Office paper, newspaper, magazines | 10 |
| Cardboard | Thin, lightweight card (cereal boxes) and corrugated cardboard | 7 |

# Molecular biology

Table S2 Samples and Golay barcodes used for Ion torrent PGM sequencing.

| **Sample ID** | **Barcode** |
| --- | --- |
| R1_0 | ACGGATCGTCAG |
| R2_0 | ACGGTGAGTGTC |
| R3_0 | ACGTACTCAGTG |
| R1_11 | ACGTCTGTAGCA |
| R2_11 | ACGTGAGAGAAT |
| R3_11 | ACGTGCCGTAGA |
| S1_0 | ACGTTAGCACAC |
| S2_0 | ACTACAGCCTAT |
| S3_0 | ACTACGTGTGGT |
| S1_11 | ACTAGCTCCATA |
| S2_11 | ACTATTGTCACG |
| S3_11 | ACTCACGGTATG |
| R+S 1_0 | ACTCAGATACTC |
| R+S 2_0 | ACTCGATTCGAT |
| R+S 3_0 | ACTCGCACAGGA |
| R+S 1_11 | ACTCTTCTAGAG |
| R+S 2_11 | ACTGACAGCCAT |
| R+S 3_11 | ACTGATCCTAGT |
| SOC1_0 | ACTGTACGCGTA |
| SOC2_0 | ACTGTCGAAGCT |
| SOC3_0 | ACTGTGACTTCA |
| SOC1_11 | ACTTGTAGCAGC |
| SOC2_11 | AGAACACGTCTC |
| SOC3_11 | AGACCGTCAGAC |

R: rumen, S: sludge, R+S: mixed inocula, SOC: Substrate Only Control. The number right after the letter corresponds at the replicate number (1,2 or 3). Each of the samples were taken at either incubation days 0 or 11.

# Statistical analyses results

Table S3 Multiple pairwise-comparison (Tukey HDS test) of the interaction effect of inocula source and initial oxygen conditions in the maximal EtOH production at initially neutral (A) and acidic (B) pH.

| **(A) Initially neutral pH**  **Inocula*Initial O_2_** | **diff** | | **lwr** | | **Upr** | | ***p*.adj** |  |
| --- | --- | --- | --- | --- | --- | --- | --- | --- |
| **Same inoculum: Aerobic – Anaerobic** | | | | | | | |  |
| Compost – Compost | 1.965 | | -8.232 | | 12.162 | | 0.999 |  |
| W. soil – W. soil | -1.025 | | -11.222 | | 9.172 | | 0.999 |  |
| C. faeces - C. faeces | -2.794 | | -12.991 | | 7.403 | | 0.996 |  |
| Rumen – Rumen | -6.764 | | -16.961 | | 3.433 | | 0.442 |  |
| Sludge – Sludge | -1.906 | | -13.306 | | 9.495 | | 0.999 |  |
| All - All | 9.514 | | -0.683 | | 19.711 | | 0.083 |  |
| **Rumen/ Initial O_2_ vs X inoculum/ Initial O_2_** | | | | | | | |  |
| Aerobic – All/ aerobic | 31.364 | | 21.167 | | 41.561 | | 0.000 |  |
| Anaerobic – All/ anaerobic | 15.086 | | 4.889 | | 25.283 | | 0.001 |  |
| Anaerobic – All/ aerobic | 24.600 | | 14.403 | | 34.797 | | 0.000 |  |
| Aerobic – Compost/ aerobic | 23.955 | | 13.758 | | 34.152 | | 0.000 |  |
| Anaerobic – Compost/ anaerobic | 15.226 | | 5.0293 | | 25.423 | | 0.001 |  |
| Anaerobic – Compost/ aerobic | 17.191 | | 6.994 | | 27.388 | | 0.000 |  |
| Aerobic – W. soil/ aerobic | 22.557 | | 12.360 | | 32.754 | | 0.000 |  |
| Anaerobic – W. soil/ anaerobic | 16.818 | | 6.6210 | | 27.015 | | 0.002 |  |
| Anaerobic – W. soil/ aerobic | 15.793 | | 5.596 | | 25.990 | | 0.001 |  |
| Aerobic – C. faeces/ aerobic | 15.193 | | 4.996 | | 25.390 | | 0.001 |  |
| Anaerobic – C. faeces/ anaerobic | 11.224 | | 1.027 | | 21.420 | | 0.023 |  |
| Anaerobic – C. faeces/ aerobic | 8.429 | | -1.7677 | | 18.626 | | 0.173* |  |
| Aerobic – Sludge/ aerobic | -27.26 | | -37.457 | | -17.063 | | 0.000 |  |
| Anaerobic – Sludge/ anaerobic | -22.402 | | -33.802 | | -11.001 | | 0.000 |  |
| Anaerobic – Sludge/ aerobic | 20.496 | | 10.299 | | 30.693 | | 0.000 |  |
| **(B) Initially acidic pH**  **Inocula*Initial oxygen** | **diff** | **lwr** | | | | **upr** | **p adj** | |
| **Same inoculum: Aerobic - Anaerobic** | | | | | | | | |
| C. faeces - C. faeces | -1.608 | -20.489 | | | | 17.273 | 0.999 | |
| Rumen - Rumen | -5.054 | -20.771 | | | | 10.665 | 0.945 | |
| Sludge - Sludge | 7.256 | -8.462 | | | | 22.974 | 0.745 | |
| All - All | -3.022 | -18.740 | | | | 12.697 | 0.997 | |
| **Sludge: treatment – Inoculum: treatment** | | | | | | | | |
| Aerobic – All: Aerobic | 15.165 | | -0.554 | 30.883 | | | 0.063 |  |
| Anaerobic – All: anaerobic | 25.442 | 9.724 | | | | 41.161 | 0.001 | |
| Anaerobic – All: aerobic | 22.421 | 6.702 | | | | 38.139 | 0.003 | |
| Aerobic – C. faeces: aerobic | 11.322 | -7.559 | | | | 30.203 | 0.467* | |
| Anaerobic – C. faeces: anaerobic | 20.186 | 1.305 | | | | 39.070 | 0.032 | |
| Anaerobic – C. faeces: aerobic | 18.578 | -0.302 | | | | 37.459 | 0.048 | |
| Aerobic – Rumen: aerobic | 16.591 | 0.872 | | | | 32.309 | 0.035 | |
| Anaerobic – Rumen: anaerobic | 28.900 | 13.182 | | | | 44.618 | 0.000 | |
| Anaerobic – Rumen: aerobic | 23.847 | 8.128 | | | | 39.565 | 0.002 | |

Where diff= difference between means of the two groups, lwr, upr= the lower and the upper values of the confidence interval at 95% and p adj= *p*-value after adjustment for the multiple comparisons. X inoculum = any inoculum apart from rumen. All= microcosms inoculated with a mixture of all inocula (see section 4.3.2).*Not significantly different.

Table S4 Mixed-source community experiment multiple pairwise-comparison (Tukey HDS test) of the interaction effect of inocula source and initial pH conditions on EtOH production.

| **Treatment** | **diff** | **lwr** | **Upr** | ***p*.adj** |
| --- | --- | --- | --- | --- |
| **Initial pH 4.5** | | | | |
| Sludge - Rumen | 12.317 | 4.399 | 20.236 | 0.001 |
| Rumen – R+S | -8.953 | -16.872 | -1.035 | 0.018 |
| Sludge - R+S | 3.364 | -4.555 | 11.282 | 0.904* |
| **Initial pH 5.5** | | | | |
| Sludge - Rumen | 13.705 | 5.787 | 21.624 | 0.000 |
| Rumen – R+S | -12.795 | -20.713 | -4.876 | 0.000 |
| Sludge - R+S | 0.910 | -7.008 | 8.829 | 1.000* |
| **Initial pH 7** | | | | |
| Sludge - Rumen | -11.564 | -19.482 | -3.645 | 0.001 |
| Rumen – R+S | 3.575 | -4.344 | 11.493 | 0.866* |
| Sludge - R+S | -7.989 | -15.908 | -0.071 | 0.047 |
| Rumen - SOC | 15.026 | 7.108 | 22.945 | 0.000 |
| Sludge - SOC | 3.462 | -4.456 | 11.381 | 0.887* |
| R+S - SOC | 11.452 | 3.533 | 19.370 | 0.002 |
| **Initial pH at maximal [EtOH]** | | | | |
| 5.5:Sludge -7:Rumen | 0.712 | -7.206 | 8.631 | 1.000* |
| 7:Rumen - 5.5:R+S | 0.198 | -7.721 | 8.116 | 1.000* |
| 5.5:Sludge - 5.5:R+S | 0.910 | -7.008 | 8.829 | 1.000* |
| **Initial pH at maximal [EtOH] vs Initial pH minimal [EtOH]: same inocula** | | | | |
| 7:Rumen - 4.5:Rumen | 17.388 | 9.469 | 25.306 | 0.000 |
| 5.5:Sludge - 7:Sludge | -12.276 | -20.195 | -4.358 | 0.001 |
| 5.5:R+S - 4.5:R+S | 8.236 | 0.318 | 16.155 | 0.037 |

Where diff= difference between means of [EtOH] between two groups, lwr, upr= the lower and upper values for 95% confidence intervals and *p* adj= *p*-value after adjustment for the multiple comparisons.

[EtOH] = EtOH concentration. *Not significantly different.
